# SRGN-mediated reactivation of the YAP/CRISPLD2 axis promotes aggressiveness of hepatocellular carcinoma

**DOI:** 10.7150/ijbs.108151

**Published:** 2025-04-28

**Authors:** Shuqiao Zhang, Hao Hu, Xinyu Li, Qiuxia Chen, Yilu Zheng, Huiting Peng, Zhuomao Mo, Wanting Hu, Yantao Li, Senkai Hong, Bijun Huang, Weihong Kuang, Shijun Zhang, Yang Cao

**Affiliations:** 1 First Affiliated Hospital of Guangzhou University of Chinese Medicine, Guangzhou University of Chinese Medicine, Guangzhou, Guangdong, China.; 2 Department of Traditional Chinese Medicine, The First Affiliated Hospital, Sun Yat-sen University, Guangzhou, Guangdong, China.; 3 Department of Hematology, The Seventh Affiliated Hospital, Sun Yat-sen University, Guangzhou, Guangdong, China.; 4 Zhejiang University Medical Center, Zhejiang University, Hangzhou, Zhejiang, China.; 5 Obstetrics & Gynecology Hospital of Fudan University, Fudan University, Shanghai, China.; 6 Sun Yat-sen University Cancer Center, Sun Yat-sen University Affiliated Tumor Hospital, Sun Yat-sen University, Guangzhou, Guangdong, China.

**Keywords:** Hepatocellular carcinoma, SRGN, Transcriptome, Tumor progression, CRISPLD2

## Abstract

Hepatocellular carcinoma (HCC) is a major contributor to global cancer-related mortality. Serglycin (SRGN) is involved in the progression of various cancers, and its overexpression is related to poor prognosis in HCC patients. Its biological role in HCC aggressiveness is unknown. This study aims to elucidate the mechanism of SRGN in HCC. Via *in vitro* and vivo experiments, we identified SRGN as a critical regulator of HCC cells migratory capability and metastasis. SRGN significantly correlated with maintaining stemness-like characteristics, emphasizing its central role in HCC progression. Mechanistically, SRGN activated YAP into the tumor cells' nucleus. Moreover, SRGN selectively upregulated CRISPLD2, establishing the SRGN/YAP/CRISPLD2 axis and promoting metastatic behavior in HCC cells. Our results revealed that CRISPLD2 is a direct target of the SRGN-mediated YAP/TEAD1 complex. SRGN orchestrated stemness maintenance, tumorigenesis, and metastasis in an autocrine way by selectively reactivating the novel YAP/CRISPLD2 axis. Besides, sorafenib with verteporfin showed a certain therapeutic effect in SRGN-positive individuals. Our work clarifies the mechanism by which SRGN promotes the invasiveness of HCC. It provides insights into targeting the SRGN-triggered signaling pathway as a potential new direction in treating HCC.

## Introduction

Hepatocellular carcinoma (HCC) is a prevalent type of malignancy that contributes substantially to the global burden of cancer mortality^1^. Although surgical removal, liver transplantation, and ablation are recognized as curative options, many HCC patients are either not eligible for surgery or face recurrence due to the cancer's local spread or the development of metastases^2^.

Proteoglycans, a class of macromolecules synthesized ubiquitously across cell types, are broadly classified into three categories, cell surface-associated proteoglycans, extracellular matrix (ECM)-secreted proteoglycans, and intracellular proteoglycans^3^. Emerging evidence underscores the critical role of proteoglycan dysregulation in tumor microenvironment remodeling and cancer pathogenesis^4^. To date, serglycin (SRGN) remains the sole characterized member of the intracellular proteoglycan subfamily that has garnered increasing attention owing to pleiotropic functions that have varying capabilities depending on the cell and immunological microenvironment.^5^, ^6^.

SRGN is a low molecular weight proteoglycan widely distributed across various cell types and can be both intracellular and integrated into the ECM^7^. The core protein of SRGN comprises one hundred fifty-eight amino acids. It is divided into three functional regions, a signal peptide, N-terminal, and C-terminal. The C-terminal region contains multiple serine and glycine repeat sequences responsible for binding to glycosaminoglycans, such as heparin, heparan sulfate, or chondroitin sulfate^5^, ^8^. SRGN is predominantly expressed in hematopoietic, endothelial, tumor, and embryonic stem cells^9^-^14^. It plays a vital role in storing and secretion of various proteases, chemokines, and cytokines^6^, ^15^. It is also implicated in immune system functions, particularly within mast cells, cytotoxic T lymphocytes, macrophages, and neutrophils^6^, ^16^-^22^. Additionally, SRGN in cancer was first discovered as a biomarker for acute myeloid leukemia and contributes to the immune evasion of tumor cells in multiple myeloma by inhibiting complement activity^23^, ^24^. SRGN regulates the migration and metastasis of head and neck squamous, nasopharyngeal, esophageal, breast, lung, and colorectal cancers through autocrine or paracrine^16^, ^25^-^29^.

Recent clinical cohort studies have demonstrated a marked overexpression of SRGN in HCC tissues. This heightened expression correlates with severe clinical outcomes, including vascular invasion, extrahepatic metastasis, and early postoperative recurrence of liver cancer. Notably, SRGN is a vital independent factor affecting HCC patient survival rates, where elevated protein levels strongly indicate a poor clinical prognosis^30^, ^31^. Although research hinted at a role for SRGN in promoting HCC aggressiveness, the specific mechanisms driving this effect are still under investigation.

In this study, we conduct a comprehensive analysis to explore the biological roles of SRGN in influencing HCC cells' aggressiveness and its related signaling pathway.

## Methods

### Processing and analysis of scRNA-seq and bulk-seq data

A full description of processing and analysis of scRNA-seq data, including data collection and quality control, cell type determination, cell subclusters metabolic analysis, copy number variation (CNV) analysis from scRNA-seq, pathway analysis, collection and calculation of functional gene module signatures and scores, cell pseudotime trajectory, cellchat, as well as collection and analysis of bulk-seq data, can be found in the Supplementary Methods.

### Serum samples

Sera samples were collected from 108 HCC patients and 17 healthy controls at the Sun Yat University Cancer Center between 2010 and 2016. The samples were collected before therapeutic procedures, such as chemotherapy and radiotherapy. This study was reviewed and approved by the Ethics Committees of the Sun Yat University Cancer Center.

### Serological detection of SRGN by ELISA

The SRGN concentration in the serum of HCC patients and the supernatant of serum-free cultured cells was measured for 48h using SRGN ELISA Kit (CUSABIO, CSB-EL022664HU) according to the manufacturer's instructions.

### Cell lines and cell culture

The human HCC cell lines LM3, MHCC-97H, SK-Hep-1, Hep3B, BEL7402, SMMC-7721, PLC/PRF/5, and LO-2 (purchased from the Shanghai Institutes for Biological Sciences, Chinese Academy of Sciences) were cultured in Dulbecco's Modified Eagle Medium (DMEM; Gibco; c11995500bt) supplemented with 10% fetal bovine serum (Invitrogen; 10099141) and penicillin (Bioss; bs-10687PA-1). Cells were maintained at 37°C in a humidified incubator with 5% CO₂. Exogenous recombinant SRGN protein were purchased from EIAab R1072h. Baicalin and daurisoline were purchased from TargetMol (USA; T3054, T2775).

### Real-time quantitative PCR

Total RNA is prepared from homogenized tissues or cells and converted to cDNA as previously described. Quantitative PCR was performed as described in Supplementary Table 1. The threshold cycles of all samples were recorded.

### Western blot

RIPA buffer was used to extract protein. Samples were centrifuged to generate supernatants, after which loading buffer was added to aliquots, and samples boiled for 10 min. Proteins were electrophoresed for two hours and transferred to PVDF membranes. A primary antibody was incubated with the membrane overnight, and a secondary antibody was added the next day. Membranes were then photographed under a fluorescence microscope.

### Cell transfection

Cells transfection was performed using siRNA-SRGN and a negative control with Lipofectamine 3000 (Invitrogen, L3000015). pQCXIH-Myc-YAP-5SA was obtained from Addgene (Catalog 33093). mRNA was extracted 48 hours post-transfection, followed by protein extraction at 72 hours.

### Cell proliferation assay

HCC cell proliferation was assessed via MTS assay. Log-phase cells were trypsinized, counted, and seeded (1,000 cells/well) in 96-well plates. Daily MTS incubation (3 hrs, 37°C) for five consecutive days was followed by absorbance measurement (490 nm). Growth curves were generated from OD values.

### Wound healing assay

A pipette tip created a gap simulating a wound when transfected cells in 6-well plates reached 85% confluence. Non-viable cells were washed away with phosphate-buffered saline (PBS), and the remaining cells were cultured in DMEM. Wound closure was monitored through microscopic imaging at 0, 24, and 48 hours.

### Migration assay

Transwell migration assays were performed with uncoated pore chambers. For migration assessment, 3×10⁴ transfected cells in 200 µL of serum-free DMEM were added to the upper chamber, while 800 µL of DMEM with 10% FBS was added to the lower chamber. After 24 hours of incubation, non-migrated cells on the upper membrane were removed with cotton swabs. Migrated cells were stained using a Diff-Quick kit following the manufacturer's protocol and counted in five random fields per chamber.

### Animal experiments

Nude mice were divided into three groups for tumor metastasis experiments. Tumor cells (1.5×10⁶) in 50 μL of PBS were injected via the tail vein. Mice were monitored twice weekly and euthanized after 8 weeks to evaluate lung metastases. Lungs were excised, paraffin-embedded, and analyzed. For tumor xenograft experiments, cells (5×10⁶) cultured under standard conditions (7.5% CO₂, 37°C) were implanted subcutaneously in 0.1 mL PBS per mouse. Tumor growth was measured biweekly with calipers, and volume was calculated. Mice were randomized into vehicle or treatment groups once tumors reached 0.2—0.4 cm^3^, and pharmacodynamic studies began at 0.5—0.8 cm^3^. Sorafenib (10 mg/kg) was administered orally daily for 21 days, while verteporfin (50 mg/kg) was given intraperitoneally every three days in a freshly prepared solution (5% PEG400, 5% Tween 80). Tumors were excised and fixed in 10% buffered formalin at specific time points. In the DEN/CCl4-induced HCC model, 15 SPF C57BL/6 male mice (23±2 g) received intraperitoneal DEN injections (25 mg/kg) for 14 days, followed by another round for 30 days to promote HCC development. HCC was established in 10 months.

### Tumor sphere analysis

Cells were seeded at a density of 1×10³ cells per well in six-well ultralow attachment plates (Corning) using MEM (Invitrogen) supplemented with N_2_ medium (Invitrogen), human EGF (10 ng/mL, Peprotech), and human bFGF (10 ng/mL, Peprotech). After 14 days of culture, the total number of spheres formed was counted.

### Flow cytometry

Add Hoechst33342 to the MHCC-97H stable knock-out SRGN and MHCC-97H cell suspension to make the final concentration of 5μg/ml; shake the cells well and put them into a constant temperature water bath at 37°C for 90 minutes. At the same time, the cell suspension should be shaken several times during the water bath, and then a low-temperature centrifuge at 4°C 1000 rpm for 10 minutes. Collect the precipitate and balance the precipitate with HBSS containing 2μg PI at 4°C to obtain the stained cells. Flow cytometry detection, excitation light is 350 nm. Acquisition wavelengths are 450 and 675, and the specimen is kept at 4°C before detection. The side population cells with weak luminescence are obtained.

### Luciferase assay

Upon achieving 80% to 95% confluence of the cultured cells, the growth medium was carefully removed, and the cells were gently washed with PBS to eliminate any detached cells and residual medium. Following removing PBS, a minimal 1× Luc-Lysis II Buffer was added to cover the cell monolayer. The plates were subsequently placed on an orbital shaker at room temperature, where they were gently rocked for 10 to 15 minutes to ensure uniform lysis of the cells. The final data were derived from the ratio of Firefly luciferase to Renilla luciferase.

### ChIP assay

Cells were cross-linked with 1% formaldehyde for 10 minutes at room temperature, followed by quenching with a solution of 10× glycine for 5 minutes. A total of 4 × 10⁶ cells were then resuspended in 200 µL of ChIP buffer supplemented with 5 µL of micrococcal nuclease and incubated at 37°C for 20 minutes. The cross-linked chromatin underwent sonication to generate DNA fragments ranging from 150 to 900 base pairs in length. Immunoprecipitation was performed utilizing 1—2 µg of YAP1 or Histone H3 (CST, D2B12) antibodies, with normal IgG as an appropriate control. ChIP-qPCR subsequently analyzed the resulting purified DNA fragments to identify sequences associated with CRISPLD2.

### Statistical analysis

Statistical analyses for this study were conducted utilizing GraphPad Prism version 8.02 and R version 4.3 software. The Wilcoxon rank-sum test was employed to compare continuous variables between two groups. The Spearman correlation method was applied to assess the correlation between gene expression. The log-rank test was used to evaluate differences in cumulative survival times. Statistical significance symbols were displayed as follows: non-significant (ns), p < 0.05 (*), p < 0.01 (**), and p < 0.001 (***).

## Results

### Single-cell RNA-seq reveals SRGN expression in HCC progression

**Figure 1:**
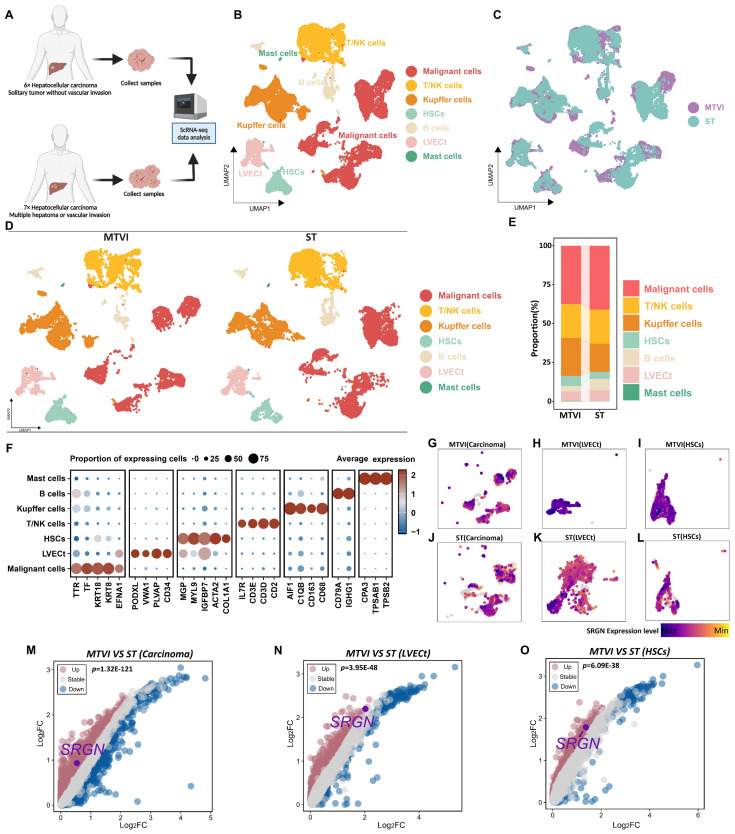
Cell landscapes of scRNA-seq analysis. **(A)** Samples for scRNA-seq analysis were taken from patients with solitary HCC status and patients with multiple hepatoma or HCC vascular invasion status. **(B)** UMAP is colored by major cell types. **(C)** UMAP is colored by cells from the ST or MTVI group. **(D)** UMAP and **(E)** stacked plots show the cell composition of the two groups. **(F)** Heatmap visualizes the marker genes for different cell types. **(G)** UMAP shows SRGN expression in malignant cells from the MTVI group. **(H)** UMAP shows SRGN expression in LVECt from the MTVI group. **(I)** UMAP shows SRGN expression in HSCs from the MTVI group. **(J)** UMAP shows SRGN expression in malignant cells from the ST group. **(K)** UMAP shows SRGN expression in LVECt from the ST group. **(L)** UMAP shows SRGN expression in HSCs from the ST group. **(M)** The volcano plot visualizes SRGN expression differences between MTVI and ST groups in malignant cells. **(N)** The volcano plot visualizes SRGN expression differences in LVECt between MTVI and ST groups. **(O)** Volcano plot visualizes SRGN expression differences in HSCs between MTVI and ST groups.

To understand the cellular composition in HCC tissues, we conducted scRNA-seq analysis on thirteen HCC patients' samples, which comprised solitary tumor conditions, tumor samples with multiple hepatoma conditions, or tumor vascular invasion conditions (Figure 1A, Supplementary Figure 1A). Following data quality control and filtering (Supplementary Figure 1B, C), we obtained single-cell transcriptomes for 68,604 cells, including 24,629 cells from solitary tumor status specimens (ST group), 43,975 cells from specimens with multiple hepatoma or tumor vascular invasion status (MTVI group).

Seven major cell types were identified according to canonical marker genes, including 9,605 T/NK cells (CD3E, CD3D, CD2, and IL7R), 9,320 Kupffer cells (CD163, CD68, C1QB, and AIF1), 2,457 B cells (CD79A and IGHG1), 2,321 hepatic stellate cells (HSCs)(MGP, MYL9, IGFBP7, ACTA2, and COL1A1), 2,888 tumor liver vascular endothelial cells (LVECt) (PODXL, VWA1, PLVAP, and CD34), 17,307 malignant cells (TTR, TF, KRT18, KRT8, and EFNA1)^32^, ^33^ and 77 mast cells (CPA3, TPSAB1, and TPSB2) (Figure 1B, F). The group-specific information of each cell population is illustrated in Figure 1C-E.

Mounting clinical evidence implicated SRGN as a critical mediator in tumor progression^23^, ^34^-^37^. Clinical data demonstrate that elevated serum SRGN levels independently correlate with diminished overall survival and accelerated HCC recurrence, and corresponding immunohistochemical analysis revealed SRGN upregulation in 56.7% of HCC patients' specimens, contrasting sharply with minimal expression observed in only 3.1% of paired non-cancerous tissues^31^. To dissect SRGN's tumor microenvironment regulatory mechanisms, we conducted a cell-type-resolved comparative analysis between localized (ST group) and advanced HCC (MTVI group). Comparative feature plots and volcano plots showed SRGN was upregulated in carcinoma cells, LVECt, and HSCs when the tumor proliferated or metastasized (Figure 1G-O).

### Dissecting the potential role of SRGN in malignant cells

**Figure 2:**
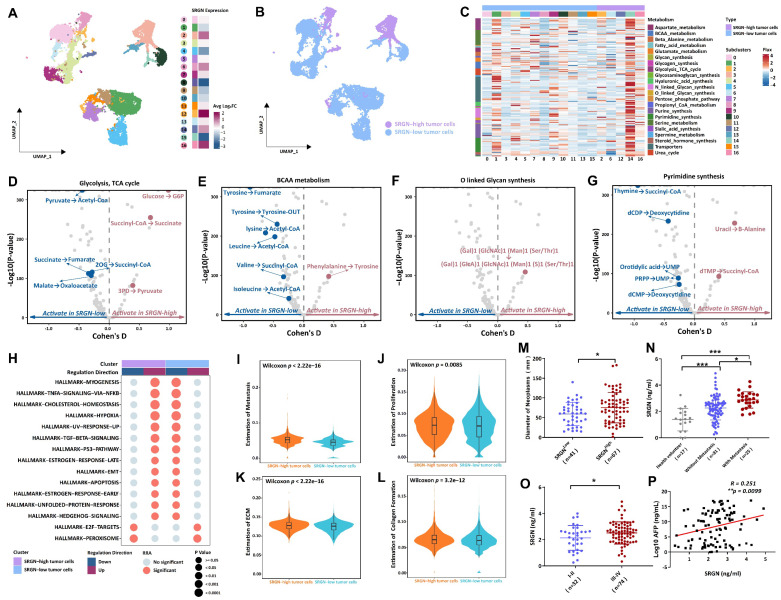
Characterization of heterogeneity between SRGN-high malignant cells and SRGN-low malignant cells. **(A)** Subcluster of malignant cells with SRGN expression. **(B)** UMAP is colored by malignant cells from the SRGN-high subcluster or SRGN-low subcluster. **(C)** Heatmap shows the metabolic flux in the SRGN-high subcluster and SRGN-low subcluster. Volcano plots display the metabolism difference of glycolysis and TCA cycle**(D)**, BCAA**(E)**, O-linked glycan synthesis**(F)**, and pyrimidine synthesis**(G)** between the two subclusters. **(H)** Dot plot shows the hallmark pathway condition in the two subclusters. Violin plots compare the metastasis score**(I)**, proliferation score**(J)**, ECM modeling score**(K)**, and collagen formation score**(L)** for two subclusters. **(M)** SRGN-high vs. SRGN-low tumor size. **(N)** SRGN sera level comparison among different HCC statuses. **(O)** Clinical stages between SRGN-high and SRGN-low patients. **(P)** SRGN expression-AFP correlation.

Given the heterogeneity in malignant cell composition, we extracted malignant cells from the ST and MTVI groups and then re-clustered malignant cells into seventeen subclusters. To explore the SRGN variation in the identified tumor cell subclusters, we computed the relative SRGN expression levels of these tumor cell subclusters. Heatmap for visualizing, the differential expression analysis revealed that five malignant cell populations exhibited high upregulated SRGN expression, including subcluster 2 (Log_2_FC=0.77, adjusted-p=2.14E-53), subcluster 6 (Log_2_FC=0.72, adjusted-p=2.12E-96), subcluster 12 (Log_2_FC=2.06, adjusted-p=2.33E-70), subcluster 14 (Log_2_FC=0.76, adjusted-p=1.89E-11), and subcluster 16 (Log_2_FC=1.63, adjusted-p= 5.28E-10) (Figure 2A, B). All p-values were adjusted using the Bonferroni method.

Metabolic diversity is known to be a significant contributor to tumor progression. We used the scFEA method to estimate malignant cell subclusters' metabolic flux to identify metabolic characteristics of the SRGN-high tumor cell population. The heatmap revealed malignant cell subclusters' average metabolic flux levels in different metabolites (Figure 2C). Further metabolic perturbation analysis indicated that the metabolic state of glycolysis, tricarboxylic acid cycle (TCA) cycle, branched-chain amino acids (BCAA) metabolism, O linked glycan synthesis, and pyrimidine synthesis were notable different between SRGN-high tumor cell population and SRGN-low cell populations (Figure 2D-G). In malignant cells characterized by high SRGN expression, within TCA cycle framework, 3-phosphoglyceric acid (3PD) was effectively converted into pyruvate, providing an essential precursor for the biosynthesis process. Succinyl-COA was more active in converting to succinate acid to combat oxidative stress, increasing the tolerance of SRGN-high tumor cells to hypoxia. Glucose was converted to glucose-6-phosphate (G6P) to enhance glycolysis for rapid energy production, fueling their rapid proliferation needs. The BCAA metabolic pathway, notably the conversion of phenylalanine to tyrosine, exhibits heightened activity in malignant cells with high SRGN expression, indicating a strategic metabolic adaptation that the cells employed to fulfill their biosynthetic demands, modulate the expression and activity of metabolic enzymes, regulate epigenetic modifications, and acclimate to the tumor microenvironment. Such metabolic fine-tuning confers a competitive advantage for SRGN-high tumor cell growth and survival. In stark contrast to malignant cells with low SRGN expression, (Gal)1 (GlcNAc)1 (Man)1 (Ser/Thr)1 to (Gal)1 (GlcA)1 (GlcNAc)1 (Man)1 (S)1 (Ser/Thr)1 in O-linked glycan synthesis was markedly enhanced in malignant cells exhibiting high levels of SRGN. The pyrimidine synthesis pathway of SRGN-high malignant cells is more active in converting uracil to β-alanine and deoxythymine nucleotide (dTMP) to succinyl-COA, which supports rapid DNA replication and cell division. Thus, in liver tumor cells, high SRGN expression might closely relate to the enhancement of metabolic reprogramming.

Results from irGSEA showed that tumor cells with elevated SRGN levels exhibit upregulation of multiple hallmark pathways, encompassing the Hedgehog signaling, unfolded protein response, early estrogen response, apoptosis, EMT, the p53 pathway, TGFβ signaling, increased ultraviolet response, hypoxia, cholesterol homeostasis, as well as the TNFA signaling pathway NFKB and myogenesis (Figure 2H). Furthermore, tumor cells presented SRGN-overexpressing displayed a higher proliferation and metastasis score, coupled with enhanced collagen formation and ECM modeling (Figure 2I-L).

Thus, we proceeded to assess the impact of SRGN levels on the invasiveness of HCC by detecting clinical serum samples. Compared to HCC patients with low SRGN levels, HCC patients with high SRGN expression had larger neoplasm diameters, accompanied by significant tumor metastasis, and were predominantly in poorer Ⅲ-Ⅳ clinical stages (Figure 2M-O). Simultaneously, we observed positive association between the serum concentrations of SRGN and Alpha-Fetoprotein (AFP) (Figure 2P). In survival analyses, HCC patients with high SRGN serum levels had lower overall survival rates than those with lower (Supplementary Figure 2A). Multivariate analysis confirmed that SRGN is an independent influencing factor correlated with poor outcomes in HCC patients (Supplementary Figure 2B). These observations align with the analysis conducted at the single-cell level, providing a coherent scenic of SRGN's role in facilitating HCC progression.

### Cellular communication of SRGN-high HCC cells with neighboring cells

**Figure 3:**
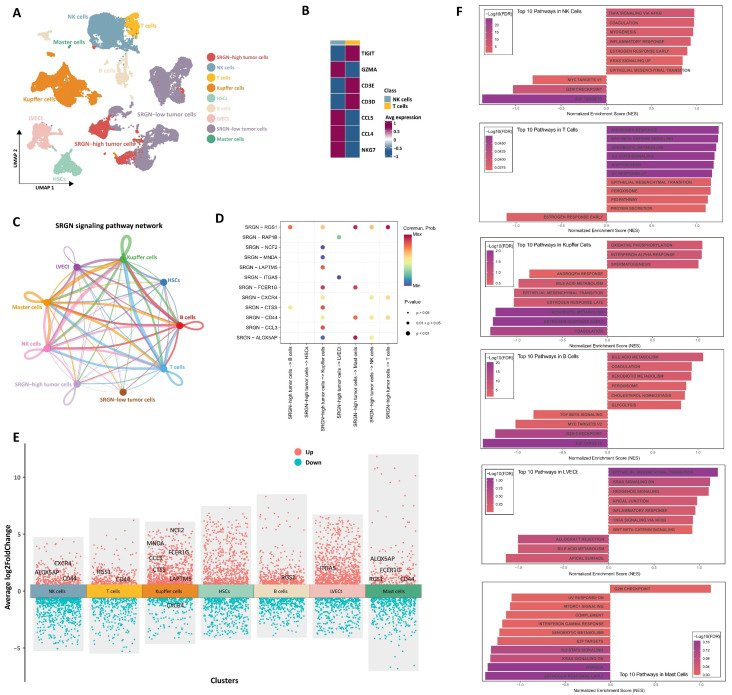
Cellular communication of SRGN-high HCC cells with neighboring cells. **(A)** Landscape of cell subpopulations in UMAP. **(B)** Genetic marker distinguishing NK and T cell populations. **(C)** SRGN-high HCC cells influence neighbor cells via the paracrine effect in the cell-cell chat network. **(D)** Analyzing receptors of neighboring cells impacted by secreted SRGN from SRGN-high HCC cells within the cell-cell communication network. **(E)** Identification of significantly differential expressed receptors in a cell communication network. **(F)** GSEA pathway analysis of SRGN-driven paracrine signaling effects in neighboring cells.

To delineate the functional influence on neighboring cells of SRGN secreted by SRGN-high HCC cell subpopulation within the tumor microenvironment, we performed CellChat analysis focusing on intercellular communication. Through genetic markers, NK/T cell subsets were classed into NK (GZMA, CCL4, CCL5, and NKG7) and T (TIGIT, CD3E, and CD3D) cell subsets (Figure 3A, B). Intercellular interactions analysis showed that HCC cells with high expression of SRGN acted on neighboring cells through the paracrine effect of SRGN (Figure 3C). Then, we analyzed the receptors of neighboring cells affected by secreted SRGN from SRGN-high HCC cells in the cell-cell communication network (Figure 3D). At the same time, we conducted a differential analysis of the expression of receptors in the cell communication network and screened out the receptors exhibiting significant responsiveness (FDR<0.05, |log2FC|>1) in recipient cells (Figure 3E). Integrated results demonstrated that SRGN secreted by SRGN-high HCC subpopulation exerted compartment-specific regulatory effects within the tumor microenvironment. Notably, NK cells exhibited enhanced expression of chemotaxis-associated receptors (CXCR4, ALOX5AP) and the adhesion mediator CD44, suggesting amplified migratory capacity. In T lymphocytes, concurrent upregulation of the signaling attenuator RGS1 and multifunctional CD44 implied dual modulation of activation thresholds and extracellular matrix interactions. Kupffer cells displayed a polarized response characterized by coordinated induction of myeloid activation markers (MNDA, CCL3, CTSS, NCF2, FCER1G, LAPTM5) alongside CXCR4 downregulation, potentially reflecting differentiation state shifts. B cell-specific RGS1 elevation pointed to altered chemokine sensing, while LVECt upregulation of ITGA5 indicated extracellular matrix adhesion remodeling. Mast cells manifested multifaceted activation through concurrent increases in inflammatory mediators (ALOX5AP, FCER1G), signaling regulators (RGS1), and migration-associated CD44, collectively suggesting SRGN-driven pro-inflammatory priming.

Leveraging differential receptor expression profiles across neighboring cell populations, we performed systematic pathway interrogation using the GSEA method to explore the functional consequences of SRGN-mediated paracrine signaling (Figure 3F). NK cells exhibited dual cell cycle restriction through downregulating E2F targets and the G2/M checkpoint, paradoxically coexisting with inflammatory activation via upregulated TNF-α/NF-κB signaling and inflammatory responses. T lymphocytes underwent metabolic-stemness reprogramming marked by WNT/β-catenin activation alongside IL2-STAT5 signaling elevation. Concurrent adipogenesis pathway upregulation and KRAS hyperactivation implied lipid metabolic rewiring might drive T cell exhaustion. Kupffer cells displayed metabolic polarization featuring enhanced oxidative phosphorylation alongside compromised xenobiotic metabolism. The coordinated interferon-α response activation and estrogen signaling inhibition suggested SRGN-driven functional redirection toward pro-inflammatory phenotypes over detoxification roles. B cell profiling revealed metabolic adaptation for antibody production, with elevated glycolytic flux and cholesterol homeostasis supporting plasma cell differentiation. This metabolic shift occurred alongside TGF-β signaling suppression, potentially unleashing inflammatory responses through IL-6 overproduction. LVECt remodeling involved endothelial plasticity programs where WNT/β-catenin activation synergized with EMT to enhance lymphatic invasive capacity. Intriguingly, KRAS signaling inhibition coexisted with TNF-α/NF-κB pathway activation, implying compensatory inflammatory mechanisms sustaining lymphangiogenesis. Mast cells demonstrated proliferative dysregulation via G2/M checkpoint upregulation paired with immune anergy, evidenced by complement system suppression and impaired interferon-γ responses. These pleiotropic effects underscore SRGN as a regulator of immune-metabolic crosstalk in HCC progression.

### SRGN promotes HCC cell migration and metastasis via autocrine

SRGN emerged as a non-redundant regulator of HCC progression, grounded in a hierarchical evidence chain through a systematic integration of single-cell multi-omics, functional pathway interrogation, and clinical validation. scRNA-seq identified SRGN as a pan-cellular marker of aggressiveness, with tumor cell subclusters, LVECt, and HSCs showing stage-dependent upregulation during metastasis, directly aligning with advanced clinical phenotypes. Beyond mere expression patterns, SRGN's functional centrality was unmasked by its orchestration of metabolically fueled plasticity—scFEA-based flux analysis revealed its role in rewiring glycolysis, TCA cycle dynamics, and pyrimidine synthesis to sustain proliferation under oxidative stress, while BCAA metabolic shifts (phenylalanine to tyrosine) indicated adaptation to nutrient-scarce microenvironments. Crucially, SRGN transcended cell-autonomous effects to dominate immune-stromal crosstalk: paracrine SRGN signaling suppressed NK cell cycling (E2F/G2-M checkpoint downregulation), polarized Kupffer cells toward pro-tumorigenic states (MNDA/CCL3↑, CXCR4↓), and primed LVECt for invasive remodeling (ITGA5↑, WNT/β-catenin activation), collectively fostering an immuno-suppressive niche permissive for metastasis. The clinical translation of these findings—serum SRGN's independent prognostic value and correlation with AFP—cemented its biological and translational relevance. By converging cell-intrinsic metabolic adaptability, microenvironmental reprogramming, and dismal patient outcomes, SRGN exemplifies a therapeutically tractable hub where molecular mechanism meets clinical urgency, rationalizing its prioritization for targeted intervention in HCC.

**Figure 4:**
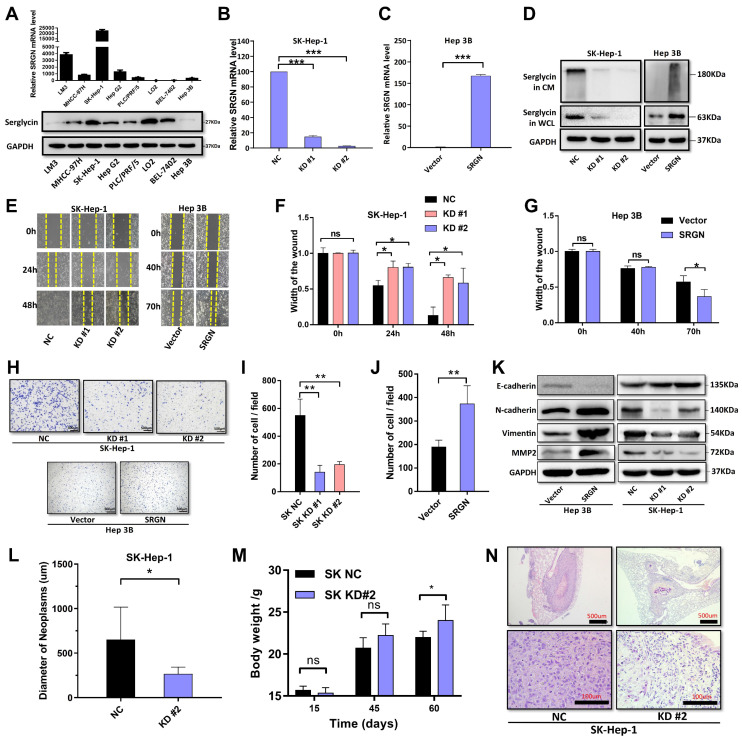
SRGN promotes HCC cell migration and metastasis. **(A)** SRGN expression profiling (qPCR/WB) in HCC cells. **(B)** SRGN knockdown efficiency validated by quantitative PCR. **(C)** SRGN overexpression confirmed by quantitative PCR. **(D)** SRGN knockdown and overexpression confirmation. **(E)** Wound-healing assay shows SRGN affects cell migration. **(F)** Quantification of wound closure rates showing significant inhibition in SRGN knockdown groups compared to controls. **(G)** Accelerated wound healing observed in SRGN-overexpressing cells relative to vector counterparts. **(H)** Transwell assay demonstrated SRGN's impact on HCC cell migration. **(I)** Statistical analysis of migrated cells showing reduction in SRGN knockdown conditions. **(J)** Quantification of Transwell assay results revealed increase in cell invasion upon SRGN overexpression. **(K)** Immunoblotting of E-cadherin, N-cadherin, Vimentin, and MMP2 at 72 hours post-transfection with SRGN suppression or overexpression. **(L)** SRGN inhibition reduced SK-Hep-1 cell metastasis *in vivo*. **(M)** Improved physiological status in SRGN knockdown groups versus controls. **(N)** Histopathological evaluation of lung tissues revealed decreased metastatic burden in SRGN-inhibited cohorts compared to controls.

To confirm the role of SRGN in promoting tumor cell migration and metastasis, we quantified its mRNA and protein levels in liver cancer cell lines using qPCR and western blot. SRGN mRNA and protein expression varied across different HCC cell lines (Figure 4A). SK-Hep-1 cells were transfected with either SRGN-specific shRNA or a negative control. Additionally, the Hep 3B cell that stably overexpressed SRGN was generated. The protein expression levels of SRGN in these modified cell lines were subsequently confirmed by q-PCR and immunoblotting assays (Figure 4B-D). We observed that SRGN knockdown reduced HCC proliferation ability, and SRGN overexpression promoted HCC proliferation (Supplementary Figure 3). Using conditioned medium form SK-Hep-1 cells KD2# group with different exogenous recombinant SRGN protein concentration via wound healing assays and Transwell test. A dose-dependent in exogenous recombinant SRGN protein concentrations was positively correlated with enhanced migratory and metastatic capacities of HCC cells, directly demonstrating SRGN's autocrine-driven role in potentiating tumor cell invasiveness and metastatic progression (Supplementary Figure 4). In wound healing assays, SK-Hep-1 cells without knockdown of SRGN showed significantly higher wound closure width than cells treated with shRNA SRGN at the same time after 24 and 48 hours, indicating that knockdown of SRGN significantly inhibited the migration ability of cells (Figure 4E, F). Correspondingly, hep 3B cells overexpressing SRHN showed accelerated wound healing, and the wound closure width reached 63% after 70 hours (Figure 4E, G). In the Transwell experiment, SK-Hep-1 cells without SRGN-knockdown showed strong migration ability within 24 hours. In contrast, SK-Hep-1 cells with SRGN knockdown significantly reduced the number of migrations, indicating that the expression of SRGN was necessary for the migration ability of cells (Figure 4H, I). Similarly, Hep 3B cells overexpressing SRGN showed enhanced migration ability (Figure 4H, J). *In vitro* analysis confirmed the expression of crucial EMT markers in HCC cell lines, including E-cadherin, N-cadherin, vimentin, and MMP2 (Figure 4K). These results indicate that SRGN promotes malignant cell migration by triggering EMT in HCC cell lines.

For *in vivo* analysis, cells with SRGN knockdown, along with their respective negative control cells, were administered via injection into the tail veins of nude mice. Following 8 weeks, the SRGN-knockdown group demonstrated significantly greater body weights than the control group (Figure 4L, Supplementary Figure 5). Subsequently, the mice were humanely euthanized, and their lungs were excised for comprehensive examination. Strikingly, the lung tissues from mice injected with SRGN-knockdown cells displayed a marked decrease in the size and quantity of metastatic nodules compared to those from the negative control (Figure 4M, N). In aggregate, these results provide evidence that SRGN significantly enhanced the metastatic capacity of malignant cells in HCC within living organisms.

### SRGN drives malignant cell stemness in HCC

**Figure 5:**
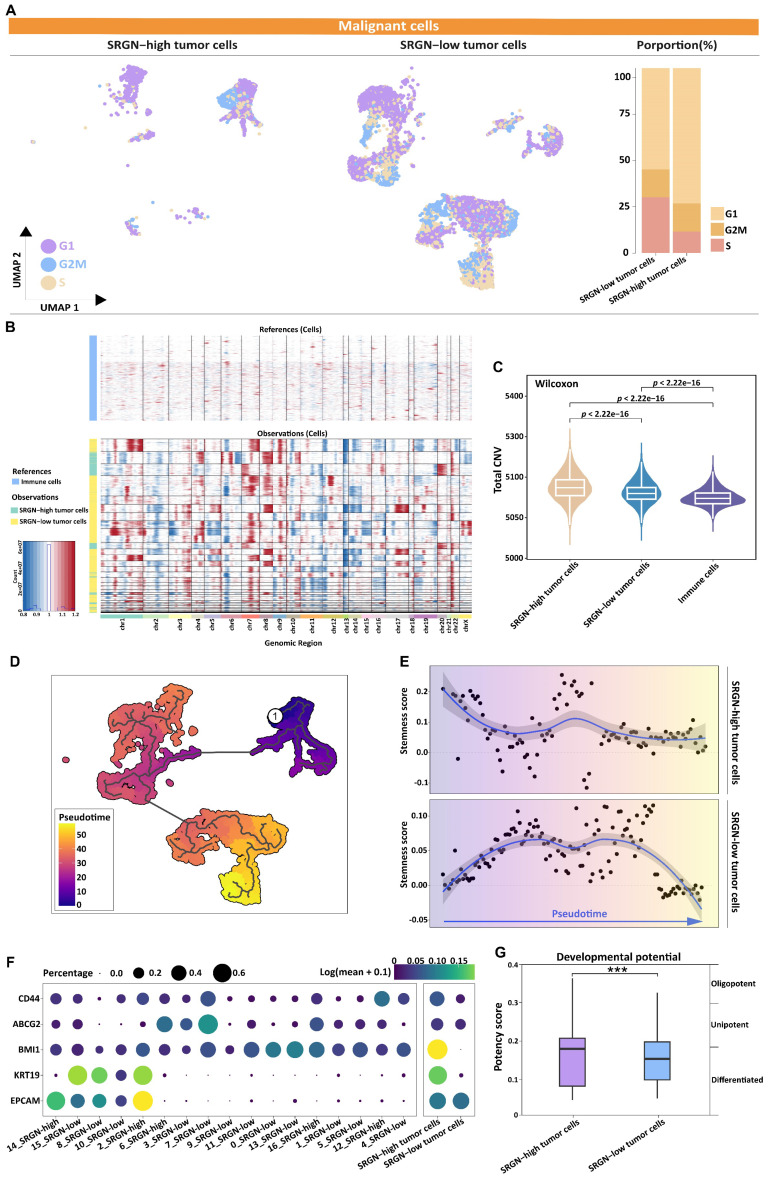
SRGN is significantly associated with stemness-like properties in HCC cells. **(A)** UMAP and stacked plot display the proportion of malignant cells in the G1, S, and G2M phases. **(B)** The heat map shows large-scale CNVs for malignant cells, with immune cells as a reference. Red represents gains, and blue represents losses. **(C)** The violin plot shows different CNV levels among SRGN-high malignant cells, SRGN-low malignant cells, and immune cells. **(D)** Pseudo-time trajectory analysis of malignant cells. Circled number one represents the trajectory root (2_SRGN-high cluster). **(E)** Dynamic variation of stemness score during the pseudo-time trajectory. **(F)** The expression of cancer stem cells related marker gene in subclusters. **(G)** Potency score comparison between SRGN-high and SRGN-low malignant cells.

We estimated the proportion of malignant cells in different cell cycle phases. As the tumor progressed, the proportion of malignant cells with high expression of SRGN gradually increased in the G1 and G2M phases (Figure 5A), validated in an external cohort (Supplementary Figure 6), which might be related to SRGN's involvement in regulating the energy and substances of these two stages of the EMT process to support DNA replication and cell division.

CNV between malignant cells was compared, using the immune cells as a reference point. Copy number variation inference of malignant cells expressing opposite levels of SRGN revealed intratumor genomic instability (Figure 5B). Tumor cells with high SRGN expression exhibited a higher rate of CNV changes, indicating significant genomic plasticity and potential activation of stemness in their cell populations (Figure 5C). Utilizing Monocle3, we delved into the intricate dynamics of cellular transitions within tumor states. Our pseudotime trajectory analysis and stemness score^38^ uncovered a lineage where SRGN-low malignant cells appeared to originate from SRGN-high malignant cells. Further, SRGN-high malignant cells maintained a comparatively higher stemness score throughout the differentiation trajectory (Figure 5D, E). Cancer stem cell-related markers, CD44, ABCG2, BMI1, and KRT19, expression in subclusters from trajectory, indicated enhanced self-renewal capacity in SRGN-high malignant cells (Figure 5F). A significant statistical variance in potency scores from Cytotrace analysis was observed between groups, with tumor cells expressing SRGN showing the highest developmental potential (Figure 5G), supporting their proliferation and differentiation capabilities, aligning with the patterns observed in pseudotime analyses.

**Figure 6:**
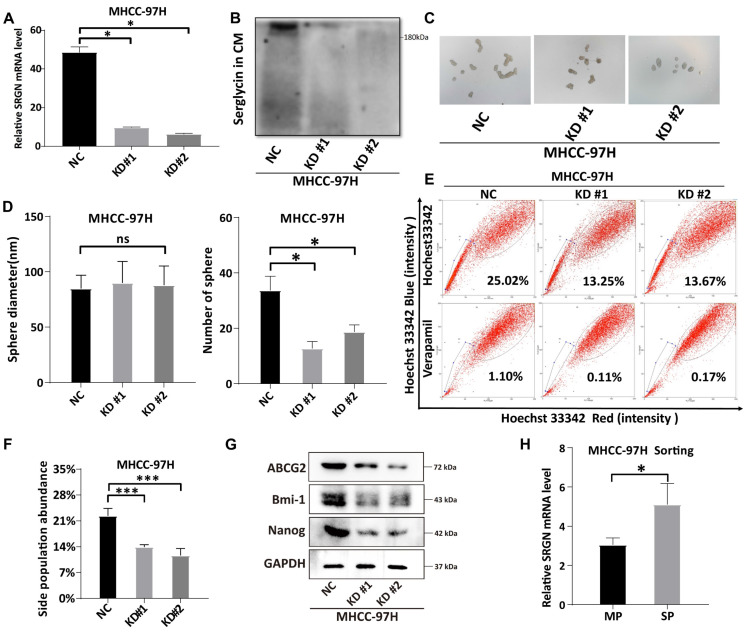
Validation of SRGN expression is related to the HCC cell's stemness characteristic. **(A, B)** Knockdown of SRGN in MHCC-97H cells verified by qPCR and immunoblotting. **(C)** The number of suspended spheres was reduced after SRGN knockdown in MHCC-97H cells. **(D)** Quantification of tumor sphere size and number. **(E, F)** Side population cell assay and abundance comparison. **(G)** Immunoblotting of cancer stem cell-related markers following SRGN knockdown in MHCC-97H cells (72 h post-transfection). **(H)** Relative SRGN mRNA levels in the main and side populations of MHCC-97H cells were determined by qPCR (normalized to GAPDH).

To investigate the role of SRGN in maintaining stemness characteristics, we knocked down SRGN in MHCC-97H cells (Figure 6A, B). SRGN knockdown significantly reduced the number of spheres formed (Figure 6C, D), highlighting its critical role in the self-renewal ability of HCC cells. Side population cells, identified by their ability to efflux the fluorescent dye Hoechst 33342, exhibit specific cancer stem cell characteristics^39^ and serve as cancer stem cell markers in HCC. Notably, SRGN knockdown in MHCC-97H cells significantly reduced the side population fraction (Figure 6E, F). Furthermore, the expression of cancer stem cell-related markers—ABCG2, Bmi-1, and Nanog—was validated, confirming that SRGN enhances the self-renewal and stemness of malignant HCC cells (Figure 6G, Supplementary Figure 7). Side population sorting assays have demonstrated that SRGN was up-regulated in cells of the side population, revealing its association with enhanced properties characteristic of cancer stem cells in HCC (Figure 6H). Collectively, these findings demonstrate that SRGN plays a pivotal role in regulating cancer stem cell-like characteristics in HCC cells.

### SRGN modulates effector YAP nuclear localization

**Figure 7:**
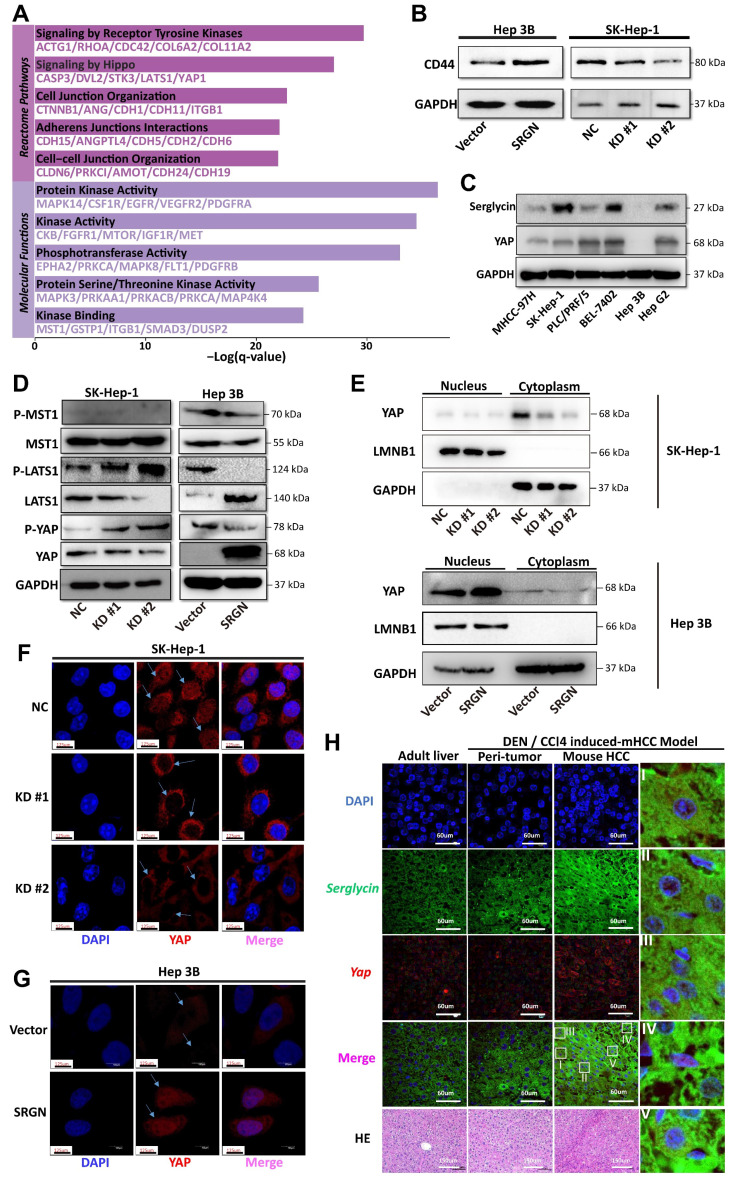
SRGN acts as a CD44 ligand to modulate YAP nuclear translocation via Hippo pathway deregulation. **(A)** Pathways enrichment analysis of SRGN-high malignant cells. **(B)** Correlation between SRGN and CD44 expression in HCC cell lines. **(C)** SRGN expression induced YAP accumulation in HCC cells as quantified by immunoblot. **(D)** Immunoblotting of MST1, P-LATS1, LATS1, P-YAP, and YAP in SRGN knockdown or overexpression conditions (72 h post-transfection). **(E)** Immunoblotting of SRGN knockdown or overexpression and YAP localization in SK-Hep-1 and Hep 3B cells. **(F, G)** Confocal immunofluorescence analysis of YAP localization in SRGN knockdown and overexpression conditions. **(H)** Confocal immunofluorescence showing SRGN and YAP localization in the DEN/CCl4-induced mouse HCC model.

Metascape enrichment analysis demonstrated significant enrichment of Hippo pathways and kinase activity in malignant cells with high SRGN expression, implying SRGN might affect tumor cell proliferation or apoptosis by regulating the Hippo signaling pathway (Figure 7A). Our prior research^40^ demonstrated that SRGN promotes the expression of CD44, leading to an enhanced capacity for self-renewal through the activation of the MAPK pathway. CD44 is a receptor for the extracellular matrix ligand SRGN. In this study, we further observed a positive correlation between SRGN and CD44 expression in malignant cells at both single-cell and bulk transcriptome levels (Supplementary Figure 8A, B) and in HCC cell lines (Figure 7B). It was presumed that the ability of SRGN to promote malignant cell invasion might be related to the YAP, a downstream signaling factor activated by CD44. Further experimental observations revealed a positive correlation between SRGN and YAP expression in HCC cell lines, supporting this hypothesis (Figure 7C). This correlation motivated us to investigate the role of SRGN in the YAP pathway further. Immunoblotting analysis revealed that SRGN overexpression reduced YAP phosphorylation (Figure 7D), suggesting that SRGN participated in the YAP pathway by inhibiting YAP phosphorylation (Figure 7E). A confocal immunofluorescence assay examining nuclear and cytosolic/membrane fractions showed reduced nuclear translocation of YAP in SK-Hep-1 cells with SRGN knockdown (Figure 7F). Conversely, SRGN overexpression in Hep 3B cells significantly increased YAP translocation to the nucleus (Figure 7G), highlighting SRGN's substantial impact on nuclear YAP expression. To further investigate the relationship between SRGN and YAP in liver cancer development, the HCC mice induced by DEN/CCl4 were used. Confocal immunofluorescence analysis demonstrated a synchronous increase in SRGN and YAP expression during HCC progression, with YAP predominantly localized in the nucleus (Figure 7H). Transwell migration assays were performed to evaluate the functional role of SRGN and its potential interaction with YAP signaling (Supplementary Figure 9A). Quantitative results revealed significant differences among experimental groups (Supplementary Figure 9B). Compared to the NC (non-knockdown control) group (653.7±63.9 cells), SRGN-depleted cells exhibited marked reductions in migration. KD#1 (336.7±48.1 cells, p=0.003 vs. NC) and KD#2 (291.0±32.8 cells, p<0.001 vs. NC) showed decreases of 48.5% and 55.5%, respectively. The more potent suppression in KD#2 versus KD#1 (p=0.041) correlated with differential SRGN knockdown efficiency. YAP5SA overexpression restored migration capacity to 517.3±5.5 cells (KD#1R, 56.9% recovery relative to KD#1; p=0.003) and 506.3±15.7 cells (KD#2R, 58.9% recovery relative to KD#2; p<0.001). Despite this rescue, both KD#1R (p=0.021 vs. NC) and KD#2R (p=0.012 vs. NC) failed to reach normal migration levels, indicating incomplete pathway restoration. KD#2R achieved greater absolute rescue than KD#1R despite more substantial initial suppression, suggesting YAP activation preferentially mitigates severe migration defects. Western blot also showed that SRGN mediated the restoration of migration ability by regulating the YAP pathway (Supplementary Figure 9C). Based on the comprehensive analysis and experimental evidence, we concluded that SRGN regulated YAP expression and nuclear localization, impacting tumor cell growth in HCC.

### CRISPLD2 serve as a downstream effector gene of the SRGN/YAP axis

**Figure 8:**
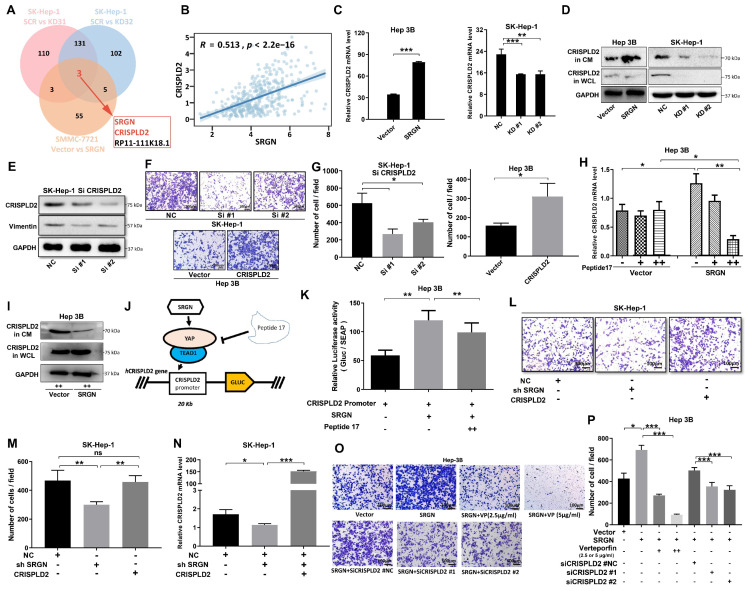
CRISPLD2 functions as a downstream effector of the SRGN/YAP axis. **(A)** SRGN and CRISPLD2 expression were overlapped among three transcriptomes groups. **(B)** Positive correlation between SRGN and CRISPLD2 expression in HCC cohorts at the bulk-seq level. **(C)** SRGN dose-dependently upregulated CRISPLD2 mRNA levels. **(D)** Immunoblot quantification confirms SRGN-induced CRISPLD2 protein elevation. **(E)** Immunoblotting shows CRISPLD2 expression in HCC cells. **(F)** CRISPLD2 knockdown reduced Transwell migration in SRGN-activated cells. **(G)** Migratory cell quantification validated CRISPLD2-dependent motility. **(H)** Peptide 17 suppressed CRISPLD2 expression in SRGN-overexpressing cells. **(I)** Immunoblot validation of CRISPLD2 protein downregulation by peptide 17 in SRGN-overexpressing cells. **(J)** Mechanistic schema of peptide 17 targeting SRGN/YAP/CRISPLD2 signaling node. **(K)** Luciferase reporter assay demonstrated CRISPLD2 promoter activity. **(L)** CRISPLD2 overexpression reversed SRGN knockdown-induced migration defect. **(M)** Quantitative migration analysis of SRGN-knockdown and CRISPLD2-overexpression. **(N)** SRGN silencing reduced CRISPLD2 transcript levels. **(O)** VP attenuated SRGN-mediated migration in dose-dependent manner. **(P)** Suppression of CRISPLD2 reduced SRGN-driven migration.

In order to investigate the downstream target of SRGN that facilitates the metastasis of HCC, RNA sequencing was conducted on the SK-Hep-1 and Hep 3B cell lines to obtain expression profiling data. Recognizing SRGN's significant role in promoting metastasis, we used microarray analysis to identify SRGN-regulated genes. From the Venn plot, CRISPLD2 emerged as a common factor in SRGN knockdown and overexpression groups (Figure 8A). At both bulk and single-cell transcriptome levels, SRGN and CRISPLD2 expression were synchronized (Figure 8B, Supplementary Figure 10A). qPCR and immunoblotting further validated the upregulation of CRISPLD2 by SRGN overexpression (Figure 8C, D; Supplementary Figure 10B, C). Utilizing small interfering RNAs to inhibit CRISPLD2 expression, we observed a significant decrease in vimentin levels, which parallels the findings obtained from SRGN knockdown experiments (Figure 8E). Cell growth assays revealed that CRISPLD2 knockdown significantly inhibited proliferation (Supplementary Figure 10D). Similarly, the Transwell assay demonstrated reduced migration capacity in cells with CRISPLD2 knockdown (Figure 8F, G), supporting the role of SRGN in promoting metastasis.

The protein interaction between the YAP and TEAD1 is essential for facilitating the oncogenic activity of YAP. Peptide 17, an inhibitor of YAP-TEAD1 interaction, suppressed CRISPLD2 expression in the SRGN-overexpressing group but had no significant effect in the vector control group (Figure 8H). Immunoblotting confirmed a significant reduction in CRISPLD2 protein levels following the administration of peptide 17 in the group with SRGN overexpression (Figure 8I). By comparing Figure 8D and Figure 8I in Hep3B cells, we observed that CRISPLD2 in SRGN-overexpressed Hep 3B cells was significantly reduced following peptide 17 intervention. To ascertain whether SRGN stimulated the expression of CRISPLD2, a luciferase reporter assay utilizing the CRISPLD2 promoter was conducted. The results indicated a significant increase in activity in response to the overexpression of SRGN. In contrast, peptide 17 intervention suppressed SRGN's regulation of CRISPLD2 activity (Figure 8J, K). Transwell assay revealed that reduced SRGN levels decreased HCC cell migration, while CRISPLD2 overexpression partially restored migration in SRGN-suppressed cells (Figure 8L, M, N; Supplementary Figure 10E).

To explore whether SRGN enhanced migration through the YAP pathway, cells were treated with verteporfin (VP) to disrupt YAP-TEAD1 complex formation. VP treatment significantly reduced preneoplastic foci and oval cell proliferation. SRGN-overexpressing cells treated with varying doses of VP exhibited reduced migration, and CRISPLD2 knockdown further inhibited cell motility (Figure 8O, P). These results uncovered that SRGN promotes HCC metastasis by upregulating CRISPLD2, a downstream effector of the SRGN/YAP pathway, which enhances HCC cell aggressiveness.

### CRISPLD2 is a novel YAP-TEAD1 target gene regulated by SRGN

**Figure 9:**
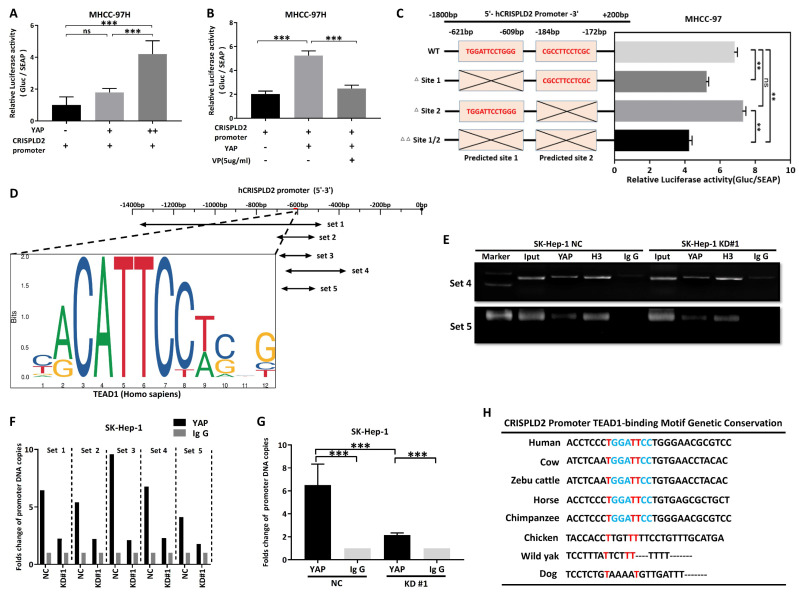
CRISPLD2 is a novel YAP-TEAD1 target gene regulated by SRGN. **(A)** Luciferase activity of the CRISPLD2 promoter in YAP-overexpressing cells. **(B)** Luciferase activity of the CRISPLD2 promoter with VP intervention in YAP-overexpressing cells. **(C)** YAP-TEAD1 binding site 1 motif in the CRISPLD2 promoter. **(D)** Schematic of CRISPLD2 promoter binding sites within the TEAD1 motif. **(E)** ChIP analysis of YAP DNA in cells stably transfected with vector or SRGN, quantified by agarose gel electrophoresis and qPCR. **(F)** Five primer pairs were used to evaluate decreased YAP DNA levels after SRGN suppression. **(G)** Fold changes in promoter DNA copy numbers. **(H)** Conserved CRISPLD2 motif sequences across species.

To confirm that SRGN modulated the downstream target CRISPLD2 within the YAP signaling pathway, we conducted transient transfection of MHCC-97H cells with the YAP. Consistent with our expectations, the luciferase reporter assay revealed a significant increase in the activity of the CRISPLD2 promoter following YAP overexpression (Figure 9A). Conversely, VP treatment suppressed CRISPLD2 promoter activity (Figure 9B), suggesting that the YAP-activated signaling pathway directly regulated the CRISPLD2 promoter.

Via the JASPAR database^41^, we identified two potential YAP-TEAD1 binding sites in the CRISPLD2 promoter region. Luciferase assays demonstrated that the elimination of binding site one resulted in a significant reduction in luciferase activity, while the deletion of binding site two did not produce any notable effect (Figure 9C). This study proved that the YAP-TEAD1 co-transcription factor facilitated the transcription of CRISPLD2 by binding to a specific sequence, referred to as site one (TGGATTCCTGGG). Chromatin immunoprecipitation analysis was performed for the subsequent validation. We designed five pairs of PCR primers targeting the CRISPLD2 promoter region (Figure 9D). The qPCR results, normalized to input levels, substantiated that YAP-TEAD1 directly bonded with the CRISPLD2 promoter, particularly at site 1, thereby regulating CRISPLD2 transcription (Figure 9E-G). Significantly, various known YAP/TEAD1 targeted genes, such as BIRC5, AREG, CCND1, CTGF, and MYC, were not activated through SRGN-mediated YAP signaling in liver cancer cell lines (Supplementary Figure 11). This observation indicated that CRISPLD2 is a novel and selective target gene of the YAP-TEAD1 complex within the SRGN-mediated HIPPO/YAP pathway. Additionally, the CRISPLD2 motif exhibited a conserved sequence across multiple species (Figure 9H).

### Combinatorial therapy targeting SRGN/YAP signaling

**Figure 10:**
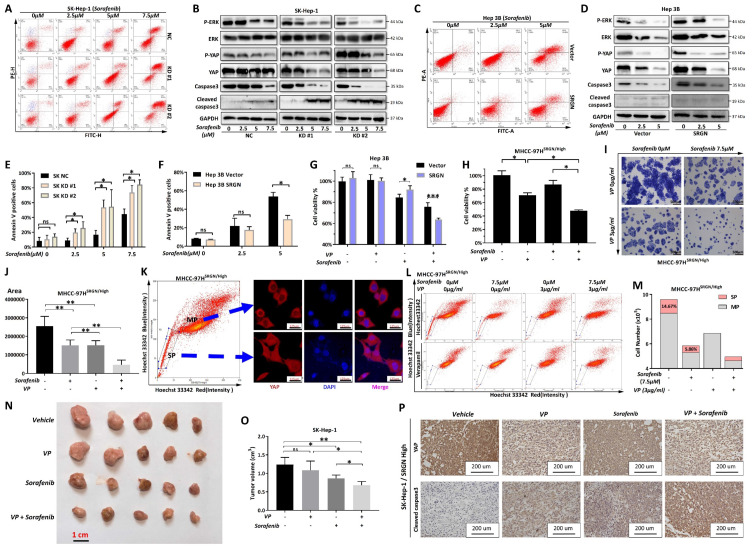
Combinatorial targeting of SRGN/YAP signaling synergizes with sorafenib to suppress HCC aggressiveness. **(A)** Flow cytometry in sorafenib-treated SK-Hep-1 cells. **(B)** Protein-level analysis of sorafenib treatment in SRGN-knockdown groups. **(C)** Flow cytometry in sorafenib-treated Hep 3B cells. **(D)** Hep 3B and Hep 3B SRGN-overexpression cells were treated with sorafenib, and analyzed by immunoblotting. **(E)** Quantification of apoptosis percentage in SK-Hep-1 cell groups. **(F)** Quantification of apoptosis percentage in Hep-3B cell groups. **(G)** VP with sorafenib reduced Hep 3B cell viability. **(H)** VP with sorafenib reduced SRGN-high MHCC-97H cells viability. **(I)** VP with sorafenib reduced SRGN-high MHCC-97H cells colony formation. **(J)** Quantification for colony formation assay. **(K)** Localization of YAP in main and side populations detected by confocal immunofluorescence. **(L)** Side population detection stained with Hoechst 33342 plus or minus verapamil. **(M)** The main population and side population cell number and percentage. **(N)** Tumor volume comparisons in SRGN-high SK-Hep-1 xenografts under different treatment schemes. **(O)** Tumor volume calculation. **(P)** IHC staining of YAP and cleaved caspase-3 in xenograft tumors under different treatment schemes.

We explored whether SRGN was directly affected by sorafenib using flow cytometry. After two days of sorafenib intervention, the percentage of apoptosis cells was measured. Comparing the apoptosis rate in the drug step-up concentration among KD#1, KD#2, and the control group, the apoptosis rate of malignant cells from the knockdown SRGN groups was much lower than that of the control group (Figure 10A, B). In the meantime, demonstrates a dose-dependent capacity to induce apoptosis in HCC cells that overexpress SRGN, compared to HCC cells with vector control (Figure 10C, D).

To examine the pathway alterations triggered by SRGN expression under sorafenib intervention, phosphorylated ERK (P-ERK), phosphorylated YAP (P-YAP), ERK, and YAP levels were analyzed. In Hep 3B cells overexpressing SRGN, sorafenib treatment dose-dependently induced caspase-3 cleavage, a hallmark of apoptosis (Figure 10E, F). Sorafenib was observed to disrupt the stimulatory effects of SRGN on P-YAP, P-ERK, YAP, and ERK, indicating targeting SRGN enhanced apoptosis via the YAP pathway. Furthermore, HCC cells that overexpressed SRGN demonstrated elevated levels of cleaved caspase-3 and decreased levels of total caspase-3 compared to the vector control group in a dose-dependent form. Additionally, cell viability assays revealed VP significantly reduced sorafenib resistance in malignant cells with high SRGN expression (Figure 10G, H). Similarly, colony formation assays showed that combining VP with sorafenib significantly suppressed the colony-forming ability of SRGN-overexpressing cells (Figure 10I, J). The confocal immunofluorescence analysis indicated YAP predominantly localized within the nuclei of cells in side population, demonstrating a significant nuclear translocation compared to the main population (Figure 10K). The side population assay demonstrated that VP significantly reduced SRGN-overexpressing malignant cells in the side population from 14.67% to 0.29%. Additionally, sorafenib reduced the main population from 10×10⁶ to 6.87×10⁶ cells (Figure 10L, M). Combining sorafenib with VP could significantly reduce the main and side populations of SRGN high-expressed malignant cells.

Moreover, utilizing the xenograft model based on SK-Hep-1 cells in nude mice, *in vivo* effects were assessed. The results demonstrated that the HCC volumes were significantly smaller in the groups receiving combination therapy compared to those treated with individual drugs (sorafenib or VP alone) (Figure 10N, O). Immunohistochemistry staining further confirmed the expression of cleaved caspase-3 and YAP in tumor samples from the *in vivo* model (Figure 10P). Comprehensively, sorafenib combined with VP could effectively eliminate SRGN high-expressed HCC cells by targeting YAP-activated tumor cell progression. We also found that HCC patients characterized by elevated SRGN expression exhibit a significantly higher potential for immune escape that is unlikely to respond favorably to anti-PD1/CTLA1 therapy (Supplementary Figure 12). Consequently, targeting the SRGN pathway in this patient population is imperative to realize meaningful therapeutic outcomes.

### Screening of potential drugs targeting SRGN protein

**Figure 11:**
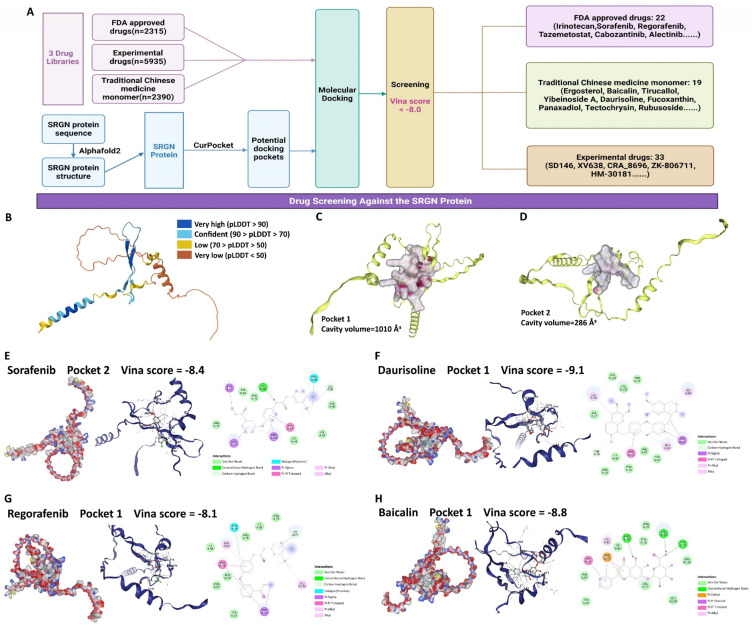
Potential medication screening for targeting SRGN protein. **(A)** Workflow of screening. **(B)** SRGN protein structure. **(C)** Docking pocket 1 of SRGN protein. **(D)** Docking pocket 2 of SRGN protein. The docking pockets and poses, and binding residues of sorafenib **(E)**, daurisoline **(F)**, regorafenib **(G)**, and baicalin **(H)** with SRGN protein.

Given the results above, SRGN protein represents a practical and promising target for developing therapies to combat HCC progression. We performed a drug screen for SRGN (Figure 11A). We extracted the latest protein structure of the SRGN using AlphaFold2, which UniProtKB reviewed manually(https://www.uniprot.org/uniprotkb/P10124/entry) (Figure 11B). Next, the predicted SRGN protein structure was subjected to cavity detection using CB-Dock2's CurPocket tool^42^, a network cavity detection method based on protein surface curvature. We screened the compounds using DrugRep^43^, an online virtual screening server based on AutoDock Vina. Using CB-Dock2's CurPocket tool, we found two pockets of SRGN protein and prepared them for docking configurations (Figure 11C, D). We screened 10,640 compounds and found 74 compounds with good binding ability to SRGN protein (the smaller the value of the score, the better the effectiveness of the docking, threshold: Vina score <-8.0), including 22 FDA-approved drugs, 23 experimental drugs, and 19 traditional Chinese medicine monomers (Supplementary Table 2). In addition, among these 74 compounds, some approved multi-targeted antitumor drugs had a high affinity for SRGN protein. The Vina binding score of sorafenib complexed with SRGN protein was -8.4 (Figure 11E). Vina's score for the regorafenib-SRGN combination was -8.1, which showed good potential as an anti-SRGN drug potential (Figure 11F). Daurisoline, an isoquinoline alkaloid isolated from the rhizome of Cortex Eucommiae, exhibited anti-inflammatory and antitumor effects^44^, and Vina score of daurisoline-SRGN complex was -9.1 (Figure 11G). Baicalin, a natural flavonoid in various medicinal plants, presented beneficial antitumor and hepatoprotective bioactivities^45^, with a vina score of -8.8 for the baicalin-SRGN complex (Figure 11H). They indicated that small medicinal plant molecules might also be potential candidates for therapeutic agents for anti-HCC progression.

**Figure 12:**
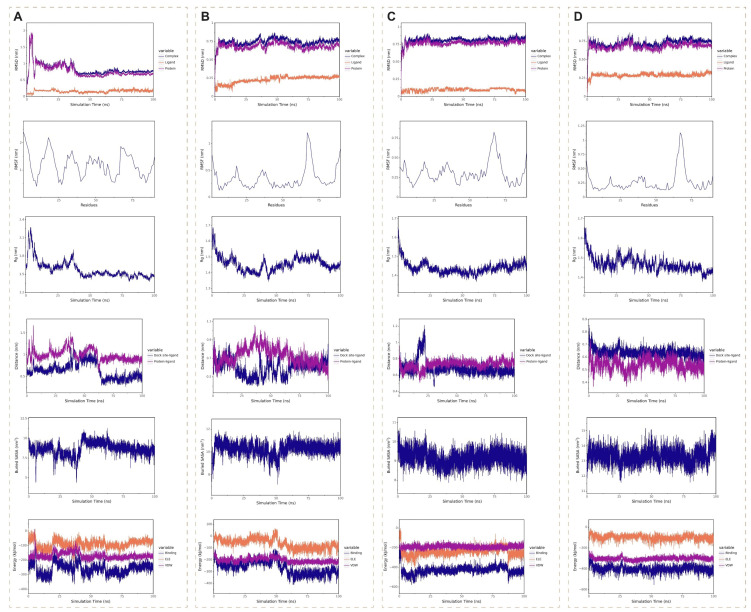
Molecular dynamics simulations of four medication monomer-SRGN complexes. **(A)** Sorafenib-SRGN complex. **(B)** Regorafenib-SRGN complex. **(C)** Baicalin-SRGN complex. **(D)** Daurisoline-SRGN complex.

Molecular dynamics simulations spanning 100 ns were performed to assess the binding stability of four SRGN complexes: sorafenib (Figure 12A), regorafenib (Figure 12B), baicalin (Figure 12C), and daurisoline (Figure 12D). The daurisoline-SRGN complex demonstrated superior stability across multiple structural and energetic parameters. The system achieved rapid equilibration, with root mean square deviation (RMSD) values stabilizing within the shortest simulation time (<5 ns) and maintaining minimal fluctuations (<0.25 Å), indicative of robust conformational stability. Root mean square fluctuation (RMSF) analysis revealed an optimal balance of rigidity and flexibility at the binding interface, suggesting adaptable yet stable ligand-protein interactions. The radius of gyration (Rg) remained consistently below 2 nm, confirming compact molecular packing and sustained binding site occupancy. Daurisoline constantly stably binds to the initial binding site of the SRGN protein. Notably, the daurisoline-SRGN complex exhibited the largest buried solvent-accessible surface area (SASA), exceeding other complexes, correlated with extensive interfacial contacts. Binding free energy calculations further highlighted its dominance. *In vitro* experiments, combined with the study of the previous dosage^46^, ^47^, daurisoline had a better inhibitory effect on the activity of SK-Hep-1 cells and a lower expression of SRGN protein than sorafenib (Supplementary Figure 13). The findings position daurisoline as a promising monomer for anti-SRGN-driven oncogenic pathways in HCC.

## Discussion

HCC poses significant therapeutic challenges due to its tendency for both intrahepatic spread and distant metastasis, along with a high likelihood of recurrence. Proteoglycans, a class of complex macromolecules rich in ECM, have been implicated in cancer progression and metastatic behavior. SRGN, a chondroitin sulfate-bearing proteoglycan initially discovered within the secretory granules of hematopoietic cells, has been observed to be readily released through exocytosis. Previously clinical studies have proved that overexpressed glycoprotein SRGN is correlated with a worse prognosis in HCC patients^30^, ^31^. However, SRGN's role in tumorigenesis and tumor progression of HCC has not been investigated. In our study, an integrative approach further reveals that highly expressed SRGN promotes aggressive phenotypes of HCC tumor cells.

At the single-cell level, our analysis revealed that the significantly elevated expression of SRGN in HCC cells was associated with multiple hepatoma conditions or when liver tumors infiltrated blood vessels, in comparison to solitary liver tumor cases. This indicates that the high expression of SRGN plays a role in the invasiveness of HCC malignant cells. Therefore, we further comprehensively analyzed the effect of high SRGN expression on HCC cells' tumor progression. Metabolic changes of tumor cells are one of the essential characteristics of tumors, which are causal to the development of tumors, making malignant cells have characteristic metabolic patterns^48^. The analysis of single-cell metabolic flux revealed significant heterogeneities in the metabolism of the glycolysis, TCA cycle, BCAA, O-linked glycan synthesis, and pyrimidine synthesis among liver malignant cells with high expression of SRGN compared to those exhibiting low expression of SRGN. The conversion of glucose to G6P in the first step of glycolysis is enhanced in HCC cells with high expression of SRGN. Previous research showed that SRGN promotes the activation of NF-κB signaling and increases glycolysis in microglia^49^. Compared with glycolysis, which mainly relies on glucose for production capacity, the tricarboxylic acid cycle can use a broader range of nutrient sources, including various amino acids and acetyl-CoA produced by lipid degradation, to produce production capacity under sufficient oxygen conditions. At the same time, many products have the function of resisting oxidative pressure. TCA enhancement is probably positively significant for tumor cell metastasis^50^. Evidence shows solid tumors' TCA rate increases during metastasis^51^. In malignant cells with high SRGN expression, 3PD is more efficiently converted to pyruvate, and succinyl CoA is catalyzed to succinate, which helps to utilize different energy sources to support their growth and proliferation, possibly providing them with additional metabolic flexibility^52^. BCAA metabolic pathway has been demonstrated to influence gene expression, protein metabolism, and the processes of apoptosis and hepatocyte regeneration^53^. In this study, the pathway of phenylalanine being catalyzed to tyrosine in SRGN-overexpressing malignant cells was active. A nested case-control study revealed that phenylalanine and tyrosine biosynthesis are significant pathways implicated in the etiology of HCC^54^. O-linked glycan synthesis is only active in HCC cells with high SRGN expression, which might potentially contribute to the EMT^55^.

Tumor cells, especially highly proliferative cells, activate the pyrimidine biosynthesis pathway to increase the supply of nucleotides to meet their needs for RNA or DNA synthesis^56^. The activity of the pyrimidine synthesis pathway in SRGN-high malignant cells reflects their rapid proliferative state. The activation of hallmark pathways, including Hedgehog signaling, unfolded protein response, EMT, hypoxia, TGFβ signaling, cholesterol homeostasis, and the TNFA signaling pathway NFKB, mirrors the highly dynamic and adaptive of tumor cells with intensive SRGN expression in their tumorigenic niche.

CellChat analysis revealed that SRGN-high HCC cells remodeled neighboring cell receptor landscapes via paracrine signaling, eliciting compartment-specific responses. In NK cells, upregulated CXCR4/CD44 and suppressed E2F/G2M checkpoints suggest enhanced migratory capacity but restricted proliferation, indicative of a terminally differentiated cytotoxic phenotype^57^-^60^. T lymphocytes exhibited metabolic-stemness reprogramming via WNT and IL2-STAT5 activation^61^. Kupffer cells displayed pro-inflammatory polarization through oxidative phosphorylation enhancement and CXCR4 downregulation, potentially altering spatial distribution in the stroma^62^, ^63^. B cells showed glycolytic metabolic adaptation for plasma differentiation and TGF-β suppression-induced IL-6 overproduction^64^, ^65^. The distinct SRGN-receptor pathway activation across cell types suggests its role as a niche organizer coordinating immuno-suppression, metabolic adaptation, and stromal remodeling.

Our study revealed that malignant cells with high expression of SRGN showed significantly enhanced activity in proliferation, migration, ECM modeling, and collagen formation compared with those with low expression, suggesting that high expression of SRGN is conducive to the invasive and progressive characteristics of HCC cells. SRGN protein, an ECM component, is higher in HCC patients than in healthy volunteers. Tumor neobiotic diameter is more extensive in HCC patients with high SRGN expression than in low-expressed patients. SRGN serum levels are higher in patients with metastatic HCC than those without metastases. The serum levels of SRGN and AFP were positively correlated, and patients with high SRGN levels had a worse survival outcome than those with low SRGN levels. These findings are consistent with previous studies^30^, ^31^.

*In vitro* and *in vivo* analyses, we first revealed that the knockdown of SRGN significantly inhibited the growth and migration of HCC cells. In contrast, the overexpression of SRGN facilitated these processes. SRGN overexpression has also been found to have the same growth-promoting effect in colon cancer cells and head and neck squamous cell carcinoma tumor cells^27^, ^28^.

In observing pseudotime trajectory from scRNA-seq, tumor cells with high expression of SRGN maintained a certain stemness during differentiation. Most Tumor cells with high SRGN expression showed unipotent differentiation potential compared to those with low SRGN expression. Known stem cell markers in HCC development, such as CD44^66^, ABCG2^67^, BMI1^68^, and EPCAM^69^, were highly expressed in SRGN-overexpressed tumor cell subgroups from various stages of differentiation. The transcriptional corepressor regulatory gene KRT19, which has recently been found to cause dedifferentiation of HCC^70^, was also highly expressed in differentiation starting point malignant cells with high SRGN expression. A series of vitro experiments revealed that SRGN expression upregulated ABCG2, BMI1, and Nanog, enhancing the sphere-forming ability of HCC cells and increasing the population of cells with cancer stem cell-like characteristics.

Some studies demonstrated that CD44 is a cell surface receptor for SRGN protein, and the binding of SRGN protein to CD44 receptor can activate several downstream signaling pathways^16^, ^49^, ^71^, ^72^. Our study consistently demonstrated that SRGN protein positively correlated with CD44 in HCC. Meanwhile, the Hippo pathway was significantly enriched in SRGN-high HCC cells. It has been determined that CD44 was an upstream regulator of YAP^73^. From vitro and vivo experiments, we found that in HCC, SRGN protein binds to CD44 to activate the Hippo/YAP signaling pathway. The Hippo/YAP pathway controls tissue growth and apoptosis in response to developmental signals, cellular contact, and density. YAP, a co-transcriptional factor of the Hippo pathway, is activated in the development and progression of HCC, which drives tumor cell survival, proliferation, invasive migration, metastasis, and stemness of liver tumor cells^74^, ^75^. Then, through comprehensive transcriptomic analysis, we found a positive correlation between SRGN and CRISPLD2 expression, indicating a potential regulatory link between them. This initial finding prompted us to delve deeper into the relationship between SRGN and CRISPLD2. The role of CRISPLD2 in HCC has yet to be observed. CRISPLD2, presently known to be involved in the work of the innate immune system, and its encoded secretory protein is rich in cysteine, which accumulates in HCC cells and contributes to their rapid proliferation and antioxidant stress^76^-^79^. In further exploration, it was found for the first time that SRGN can regulate CRISPLD2 expression through the YAP axis and positively promote tumor cell proliferation. YAP/TEAD1 is a co-transcription factor of CRISPLD2. This finding is particularly intriguing because both CRISPLD2 and YAP pathways were known to be associated with embryonic development^80^-^83^. In SRGN-mediated HCC cells, the YAP/CRISPLD2 axis was selectively reactivated, setting it apart from the activation of other known YAP pathway target genes. The above evidence demonstrates a unique invasive-promoting role for the SRGN/YAP/CRISPLD2 axis in HCC cells, potentially offering new insights into the molecular mechanisms underlying tumorigenesis and novel therapeutic strategies.

Sorafenib is a frontline used medication for advanced metastatic liver cancer^84^, and combined with VP. It may surmount chemo-insensitivity stemming from the passive lysosomal sequestration of anti-cancer drugs^85^, so we also explored their effect on SRGN-mediated tumor progression. Our findings revealed that the synergistic administration of sorafenib and VP exerted notable anti-SRGN-high tumor cell effects *in vivo* and *in vitro*. Sorafenib plus VP dramatically reduced the tumor size and caner stem cells. Massive molecular docking and dynamics simulations revealed daurisoline, a natural isoquinoline alkaloid, as a novel potential SRGN-targeting inhibitor in HCC^46^. These findings offer promising novel insights for underlying individual precision treatment in SRGN-positive HCC and hold potential therapeutic strategies for other types of cancer.

## Conclusion

In conclusion, our work confirms that SRGN promotes the invasiveness of HCC. SRGN supports the proliferation and metastatic potential and sustains stemness characteristics of HCC cells via the autocrine activation of the YAP/CRISPLD2 signaling pathway. Our findings provide insights into targeting SRGN-triggered signaling as a promising strategy for reversing tumor therapeutic resistance. Therapeutic approaches targeting SRGN may be a new direction in treating HCC.

## Supplementary Material

Supplementary methods, figures and tables.

## Abbreviations

HCC: Hepatocellular carcinoma
SRGN: Serglycin
Ns: non-significant
scRNA-seq: Single-cell RNA sequencing
CNV: Copy number variation
PBS: Phosphate-buffered saline
ST: Solitary HCC status
MTVI: Multiple hepatoma or HCC vascular invasion status
HSCs: Hepatic stellate cells
LVECt: tumor liver vascular endothelial cells
TCA: Tricarboxylic acid cycle
BCAA: Branched-chain amino acids
3PD: 3-phosphoglyceric acid
G6P: Glucose-6-phosphate
dTMP: Deoxythymine nucleotide
AFP: Alpha-Fetoprotein
NC: Non-knockdown control
KD: Knockdown
CM: Cell membrane
WCL: Whole cell lysate
RMSD: Root mean square deviation
RMSF: Root mean square fluctuation
Rg: Radius of gyration
SASA: solvent-accessible surface area

## References

[1] Sung H, Ferlay J, Siegel RL (2021). Global Cancer Statistics 2020: GLOBOCAN Estimates of Incidence and Mortality Worldwide for 36 Cancers in 185 Countries. CA Cancer J Clin.

[2] Bruix J, Reig M, Sherman M (2016). Evidence-Based Diagnosis, Staging, and Treatment of Patients with Hepatocellular Carcinoma. Gastroenterology.

[3] Theocharis AD, Skandalis SS, Tzanakakis GN (2010). Proteoglycans in health and disease: novel roles for proteoglycans in malignancy and their pharmacological targeting. FEBS J.

[4] Iozzo RV, Sanderson RD (2011). Proteoglycans in cancer biology, tumour microenvironment and angiogenesis. J Cell Mol Med.

[5] Scully OJ, Chua PJ, Harve KS (2012). Serglycin in health and diseases. Anat Rec (Hoboken).

[6] Kolset SO, Pejler G (2011). Serglycin: a structural and functional chameleon with wide impact on immune cells. J Immunol.

[7] Toyama-Sorimachi N, Sorimachi H, Tobita Y (1995). A novel ligand for CD44 is serglycin, a hematopoietic cell lineage-specific proteoglycan. Possible involvement in lymphoid cell adherence and activation. J Biol Chem.

[8] Kolset SO, Tveit H (2008). Serglycin-structure and biology. Cell Mol Life Sci.

[9] Elliott JF, Miller CL, Pohajdak B (1993). Induction of a proteoglycan core protein mRNA in mouse T lymphocytes. Mol Immunol.

[10] Kulseth MA, Mustorp SL, Uhlin-Hansen L (1998). Serglycin expression during monocytic differentiation of U937-1 cells. Glycobiology.

[11] Maillet P, Alliel PM, Mitjavila MT (1992). Expression of the serglycin gene in human leukemic cell lines. Leukemia.

[12] Schick BP, Gradowski JF, San Antonio JD (2001). Synthesis, secretion, and subcellular localization of serglycin proteoglycan in human endothelial cells. Blood.

[13] Schick BP, Ho HC, Brodbeck KC (2003). Serglycin proteoglycan expression and synthesis in embryonic stem cells. Biochim Biophys Acta.

[14] Toyama-Sorimachi N, Kitamura F, Habuchi H (1997). Widespread expression of chondroitin sulfate-type serglycins with CD44 binding ability in hematopoietic cells. J Biol Chem.

[15] Kolseth IB, Reine TM, Vuong TT (2015). Serglycin is part of the secretory repertoire of LPS-activated monocytes. Immun Inflamm Dis.

[16] Guo JY, Chiu CH, Wang MJ (2020). Proteoglycan serglycin promotes non-small cell lung cancer cell migration through the interaction of its glycosaminoglycans with CD44. J Biomed Sci.

[17] Dressel R, Raja SM, Honing S (2004). Granzyme-mediated cytotoxicity does not involve the mannose 6-phosphate receptors on target cells. J Biol Chem.

[18] Galvin JP, Spaeny-Dekking LH, Wang B (1999). Apoptosis induced by granzyme B-glycosaminoglycan complexes: implications for granule-mediated apoptosis *in vivo*. J Immunol.

[19] Metkar SS, Wang B, Aguilar-Santelises M (2002). Cytotoxic cell granule-mediated apoptosis: perforin delivers granzyme B-serglycin complexes into target cells without plasma membrane pore formation. Immunity.

[20] Raja SM, Metkar SS, Honing S (2005). A novel mechanism for protein delivery: granzyme B undergoes electrostatic exchange from serglycin to target cells. J Biol Chem.

[21] Raja SM, Wang B, Dantuluri M (2002). Cytotoxic cell granule-mediated apoptosis. Characterization of the macromolecular complex of granzyme B with serglycin. J Biol Chem.

[22] Veugelers K, Motyka B, Goping IS (2006). Granule-mediated killing by granzyme B and perforin requires a mannose 6-phosphate receptor and is augmented by cell surface heparan sulfate. Mol Biol Cell.

[23] Niemann CU, Kjeldsen L, Ralfkiaer E (2007). Serglycin proteoglycan in hematologic malignancies: a marker of acute myeloid leukemia. Leukemia.

[24] Theocharis AD, Seidel C, Borset M (2006). Serglycin constitutively secreted by myeloma plasma cells is a potent inhibitor of bone mineralization *in vitro*. J Biol Chem.

[25] Zhang Z, Deng Y, Zheng G (2017). SRGN-TGFbeta2 regulatory loop confers invasion and metastasis in triple-negative breast cancer. Oncogenesis.

[26] Li XJ, Ong CK, Cao Y (2011). Serglycin is a theranostic target in nasopharyngeal carcinoma that promotes metastasis. Cancer Res.

[27] Xie J, Qi X, Wang Y (2021). Cancer-associated fibroblasts secrete hypoxia-induced serglycin to promote head and neck squamous cell carcinoma tumor cell growth *in vitro* and *in vivo* by activating the Wnt/beta-catenin pathway. Cell Oncol (Dordr).

[28] Xu Y, Xu J, Yang Y (2018). SRGN Promotes Colorectal Cancer Metastasis as a Critical Downstream Target of HIF-1alpha. Cell Physiol Biochem.

[29] Zhu Y, Lam AKY, Shum DKY (2021). Significance of serglycin and its binding partners in autocrine promotion of metastasis in esophageal cancer. Theranostics.

[30] He J, Zeng ZC, Xiang ZL (2014). Mass spectrometry-based serum peptide profiling in hepatocellular carcinoma with bone metastasis. World J Gastroenterol.

[31] He L, Zhou X, Qu C (2013). Serglycin (SRGN) overexpression predicts poor prognosis in hepatocellular carcinoma patients. Med Oncol.

[32] Li K, Zhang R, Wen F (2024). Single-cell dissection of the multicellular ecosystem and molecular features underlying microvascular invasion in HCC. Hepatology.

[33] Puram SV, Tirosh I, Parikh AS (2017). Single-Cell Transcriptomic Analysis of Primary and Metastatic Tumor Ecosystems in Head and Neck Cancer. Cell.

[34] Oynebraten I, Hansen B, Smedsrod B (2000). Serglycin secreted by leukocytes is efficiently eliminated from the circulation by sinusoidal scavenger endothelial cells in the liver. J Leukoc Biol.

[35] Grover A, Edwards SA, Bourdon M (1987). Proteoglycan-19, laminin and collagen type IV production is correlated with the levels of mRNA in F9 cell aggregates differentiating in the presence or absence of cyclic AMP. Differentiation.

[36] Gasimli L, Stansfield HE, Nairn AV (2013). Structural remodeling of proteoglycans upon retinoic acid-induced differentiation of NCCIT cells. Glycoconj J.

[37] Korpetinou A, Skandalis SS, Labropoulou VT (2014). Serglycin: at the crossroad of inflammation and malignancy. Front Oncol.

[38] Desert R, Rohart F, Canal F (2017). Human hepatocellular carcinomas with a periportal phenotype have the lowest potential for early recurrence after curative resection. Hepatology.

[39] Xu L, Huang TJ, Hu H (2018). The developmental transcription factor IRF6 attenuates ABCG2 gene expression and distinctively reverses stemness phenotype in nasopharyngeal carcinoma. Cancer Lett.

[40] Chu Q, Huang H, Huang T (2016). Extracellular serglycin upregulates the CD44 receptor in an autocrine manner to maintain self-renewal in nasopharyngeal carcinoma cells by reciprocally activating the MAPK/beta-catenin axis. Cell Death Dis.

[41] Rauluseviciute I, Riudavets-Puig R, Blanc-Mathieu R (2024). JASPAR 2024: 20th anniversary of the open-access database of transcription factor binding profiles. Nucleic Acids Res.

[42] Liu Y, Yang X, Gan J (2022). CB-Dock2: improved protein-ligand blind docking by integrating cavity detection, docking and homologous template fitting. Nucleic Acids Res.

[43] Gan JH, Liu JX, Liu Y (2023). DrugRep: an automatic virtual screening server for drug repurposing. Acta Pharmacol Sin.

[44] Xue L, Liu P (2021). Daurisoline inhibits hepatocellular carcinoma progression by restraining autophagy and promoting cispaltin-induced cell death. Biochem Biophys Res Commun.

[45] Sun J, Yang X, Sun H (2023). Baicalin inhibits hepatocellular carcinoma cell growth and metastasis by suppressing ROCK1 signaling. Phytother Res.

[46] Zhang X, Zhang JG, Mu W (2021). The role of daurisoline treatment in hepatocellular carcinoma: Inhibiting vasculogenic mimicry formation and enhancing sensitivity to sorafenib. Phytomedicine.

[47] Zhou X, Luo J, Xie H (2022). MCM2 promotes the stemness and sorafenib resistance of hepatocellular carcinoma cells via hippo signaling. Cell Death Discov.

[48] Martinez-Reyes I, Chandel NS (2021). Cancer metabolism: looking forward. Nat Rev Cancer.

[49] Qian Y, Yang L, Chen J (2024). SRGN amplifies microglia-mediated neuroinflammation and exacerbates ischemic brain injury. J Neuroinflammation.

[50] Cai Z, Li CF, Han F (2020). Phosphorylation of PDHA by AMPK Drives TCA Cycle to Promote Cancer Metastasis. Mol Cell.

[51] Bartman CR, Weilandt DR, Shen Y (2023). Slow TCA flux and ATP production in primary solid tumours but not metastases. Nature.

[52] Wang C, Ren J, Zhou L (2019). An Aldolase-Catalyzed New Metabolic Pathway for the Assimilation of Formaldehyde and Methanol To Synthesize 2-Keto-4-hydroxybutyrate and 1,3-Propanediol in Escherichia coli. ACS Synth Biol.

[53] Tajiri K, Shimizu Y (2013). Branched-chain amino acids in liver diseases. World J Gastroenterol.

[54] Li ZY, Shen QM, Wang J (2024). Prediagnostic plasma metabolite concentrations and liver cancer risk: a population-based study of Chinese men. EBioMedicine.

[55] Beaman EM, Carter DRF, Brooks SA (2022). GALNTs: master regulators of metastasis-associated epithelial-mesenchymal transition (EMT)?. Glycobiology.

[56] Lafita-Navarro MC, Venkateswaran N, Kilgore JA (2020). Inhibition of the de novo pyrimidine biosynthesis pathway limits ribosomal RNA transcription causing nucleolar stress in glioblastoma cells. PLoS Genet.

[57] Gholami S, Baca Y, Brodskiy P (2022). CXCR4 overexpression: An indicator of poor survival and predictor of response to immunotherapy in patients with metastatic colorectal cancer. J clin oncol.

[58] Ames E, Canter RJ, Grossenbacher SK (2015). NK Cells Preferentially Target Tumor Cells with a Cancer Stem Cell Phenotype. J Immunol.

[59] Streltsova MA, Ustiuzhanina MO, Barsov EV (2021). Telomerase Reverse Transcriptase Increases Proliferation and Lifespan of Human NK Cells without Immortalization. Biomedicines.

[60] Jung H, Hsiung B, Pestal K (2012). RAE-1 ligands for the NKG2D receptor are regulated by E2F transcription factors, which control cell cycle entry. J Exp Med.

[61] Raynor JL, Chapman NM, Chi H (2021). Metabolic Control of Memory T-Cell Generation and Stemness. Cold Spring Harb Perspect Biol.

[62] Vrba J, Modriansky M (2002). Oxidative burst of Kupffer cells: target for liver injury treatment. Biomed Pap Med Fac Univ Palacky Olomouc Czech Repub.

[63] Ferre N, Claria J (2006). New insights into the regulation of liver inflammation and oxidative stress. Mini Rev Med Chem.

[64] Molnarfi N, Bjarnadottir K, Benkhoucha M (2017). Activation of human B cells negatively regulates TGF-beta1 production. J Neuroinflammation.

[65] Imahashi N, Basar R, Huang Y (2022). Activated B cells suppress T-cell function through metabolic competition. J Immunother Cancer.

[66] Ji J, Wang XW (2012). Clinical implications of cancer stem cell biology in hepatocellular carcinoma. Semin Oncol.

[67] Yang XR, Xu Y, Yu B (2010). High expression levels of putative hepatic stem/progenitor cell biomarkers related to tumour angiogenesis and poor prognosis of hepatocellular carcinoma. Gut.

[68] Chiba T, Seki A, Aoki R (2010). Bmi1 promotes hepatic stem cell expansion and tumorigenicity in both Ink4a/Arf-dependent and -independent manners in mice. Hepatology.

[69] Terris B, Cavard C, Perret C (2010). EpCAM, a new marker for cancer stem cells in hepatocellular carcinoma. J Hepatol.

[70] Han S, Fan H, Zhong G (2024). Nuclear KRT19 is a transcriptional corepressor promoting histone deacetylation and liver tumorigenesis. Hepatology.

[71] Guo JY, Hsu HS, Tyan SW (2017). Serglycin in tumor microenvironment promotes non-small cell lung cancer aggressiveness in a CD44-dependent manner. Oncogene.

[72] Guo J-Y, Tyan S-W, Hsu H-S (2015). Secreted serglycin in tumor microenvironment promotes tumor malignancies in a CD44-dependent manner. Cancer Research.

[73] Fan Z, Xia H, Xu H (2018). Standard CD44 modulates YAP1 through a positive feedback loop in hepatocellular carcinoma. Biomed Pharmacother.

[74] Thompson BJ (2020). YAP/TAZ: Drivers of Tumor Growth, Metastasis, and Resistance to Therapy. Bioessays.

[75] Qing J, Ren Y, Zhang Y (2022). Dopamine receptor D2 antagonism normalizes profibrotic macrophage-endothelial crosstalk in non-alcoholic steatohepatitis. J Hepatol.

[76] Yamauchi T, Okano Y, Terada D (2024). Epigenetic repression of de novo cysteine synthetases induces intra-cellular accumulation of cysteine in hepatocarcinoma by up-regulating the cystine uptake transporter xCT. Cancer Metab.

[77] Yuan Q, Chiquet BT, Devault L (2012). Craniofacial abnormalities result from knock down of nonsyndromic clefting gene, crispld2, in zebrafish. Genesis.

[78] Gibbs GM, Roelants K, O'Bryan MK (2008). The CAP superfamily: cysteine-rich secretory proteins, antigen 5, and pathogenesis-related 1 proteins-roles in reproduction, cancer, and immune defense. Endocr Rev.

[79] Gaikwad AS, Hu J, Chapple DG (2020). The functions of CAP superfamily proteins in mammalian fertility and disease. Hum Reprod Update.

[80] Zhang H, Sweezey NB, Kaplan F (2015). LGL1 modulates proliferation, apoptosis, and migration of human fetal lung fibroblasts. Am J Physiol Lung Cell Mol Physiol.

[81] Zhang H, Kho AT, Wu Q (2016). CRISPLD2 (LGL1) inhibits proinflammatory mediators in human fetal, adult, and COPD lung fibroblasts and epithelial cells. Physiol Rep.

[82] Lian I, Kim J, Okazawa H (2010). The role of YAP transcription coactivator in regulating stem cell self-renewal and differentiation. Genes Dev.

[83] Chung H, Lee BK, Uprety N (2016). Yap1 is dispensable for self-renewal but required for proper differentiation of mouse embryonic stem (ES) cells. EMBO Rep.

[84] Komatsu S, Yano Y, Mimura T (2024). Current Status of Conversion Hepatectomy After Sorafenib and Lenvatinib Treatment for Unresectable Hepatocellular Carcinoma. Anticancer Res.

[85] Gavini J, Dommann N, Jakob MO (2019). Verteporfin-induced lysosomal compartment dysregulation potentiates the effect of sorafenib in hepatocellular carcinoma. Cell Death Dis.

