# Supplementary material for: SRGN-mediated reactivation of the YAP/CRISPLD2 axis promotes aggressiveness of hepatocellular carcinoma
Source: Int J Biol Sci. 2025 Apr 28;21(7):3262–85. doi: 10.7150/ijbs.108151 (PMC12080381; doi:10.7150/ijbs.108151)
Supplement: Supplementary file 1 — Supplementary methods, figures and tables. [file ijbsv21p3262s1.pdf]

SRGN-mediated reactivation of the YAP/CRISPLD2 axis promotes aggressiveness of hepatocellular carcinoma

Shuqiao Zhang[1*]; Hao Hu[1*]; Xinyu Li[1*]; Qiuxia Chen[2*]; Yilu Zheng[3]; Huiting Peng[1]; Zhuomao Mo[4]; Wanting Hu[5]; Yantao Li[1]; Senkai Hong[1]; Bijun Huang[6#]; Weihong Kuang[1#]; Shijun Zhang[2#]; Yang Cao[1#]

Supplementary Materials Contents:

1. Supplementary methods
2. Supplementary figures
3. Supplementary tables
4. Supplementary references

Supplementary methods:

Data collection

We procured single-cell transcriptomic datasets GSE151530, GSE166635, and GSE149614 from the Gene Expression Omnibus repository (GEO, https://www.ncbi.nlm.nih.gov/geo). At the bulk transcriptomic level, mRNA expression profiles, and clinical data of HCC patients were retrieved from The Cancer Genome Atlas Liver Hepatocellular Carcinoma (LIHC) project via the UCSC Xena platform(http://xena.ucsc.edu/), GSE124751 from the GEO, HCCDB18 from HCCDB (http://lifeome.net:809/#/download). Additionally, pertinent metadata were extracted from the original research.

ScRNA-seq data processing

With its default settings, the Seurat workflow version 5.0 was utilized to analyze scRNA-seq data. Initial quality control steps were taken to filter out cells with a low gene detection count (fewer than 500 genes) and an elevated mitochondrial gene expression rate (over 30%). Following the quality control phase, scTransform was applied for data normalization, accounting for mitochondrial gene expression levels, Unique Molecular Identifiers (UMIs), and gene detection counts. Principal Component Analysis (PCA) was conducted utilizing the RunPCA function to achieve dimensionality reduction in the dataset. Subsequently, Uniform Manifold Approximation and Projection (UMAP) was employed through the RunUMAP function to further enhance data visualization by reducing dimensionality. Clustering analysis was performed using the FindClusters function, with a resolution parameter set at 0.2, and the Louvain method was applied to optimize modularity. To identify cell types, we compiled a set of lineage-specific markers to distinguish the primary cell populations. These included CD3E, CD3D, CD2, and IL7R for T/NK cells; CD163, CD68, C1QB, and AIF1 for Kupffer cells; CD79A and IGHG1 for B cells; MGP, MYL9, IGFBP7, ACTA2, and COL1A1 for hepatic stellate cells; PODXL, VWA1, PLVAP, and CD34 for tumor liver vascular endothelial cells; TTR, TF, KRT18, KRT8, and EFNA1 for malignant cells; and CPA3, TPSAB1, and TPSB2 for mast cells. The AddModuleScore function within Seurat was leveraged to compute proliferation[1], metastasis[2], extracellular matrix (ECM) modeling[3], and collagen formation[4] scores for subcluster cells. We calculated cell cycle phase scores utilizing the CellCycleScoring function in conjunction with a carefully curated list of canonical markers derived from Seurat. This classification allowed us to categorize cells into G1, S, or G2M phases based on their cell cycle status.

Single-cell metabolic analysis

ScFEA is a metabolic flux relative rate evaluation software based on the Flux Balance algorithm[5]. It can evaluate the changes of enzymes and transporters through scRNA-seq data expression changes, combined with flux balance constraint probability. According to the human KEGG metabolic map[6], key metabolic modules were obtained. Establish flux balance constraint probability model, neural networks optimization solver and multi-layer neural networks model to obtain complex cascade information from transcriptome to metabolome. Define a loss function to infer the cellular metabolic flux of scRNA-seq data. The t-test and Cohen's formula were used to determine the statistical significance of metabolic differences between groups and to assess the degree of differences.

Single-cell rank-based gene set enrichment analysis

We analyzed the hallmark pathways' activity within malignant cell subpopulations using irGSEA[7]. This software consolidates multiple enrichment analysis techniques on scRNA-seq levels. The integrated methods encompass AUCell[8], VISION[9], GSVA[10], singscore[11], ssGSEA[12], JASMINE[13], and viper (https://github.com/alevax/pyviper). The robust rank aggregation algorithm[14] was applied to pinpoint gene sets consistently enriched across these various analytical approaches. Thereby, irGSEA provided a comprehensive view of the activity of crucial pathways within the malignant cell subpopulations.

InferCNV for scRNA-seq data analysis

InferCNV tool (https://github.com/broadinstitute/inferCNV) calculated large-scale copy number variations (CNVs) from scRNA-seq data. T/NK cells (immune cells) served as spiked-in controls (reference). We utilized the InfercnvObject function to compile a raw scRNA-seq counts matrix, a scRNA-seq annotation file, and a file detailing the positions of genes and chromosomes (https://www.gencodegenes.org/). The InferCNV analysis was executed with a stringent cutoff value set at 0.1 to ensure the reliability of the inferred CNVs.

Single-cell trajectory construction

The cell trajectory was established using Monocle3 (https://cole-trapnell-lab.github.io/monocle3/). For pseudotime analysis, we used the UMAP coordinates to plot a graph that delineates the path of cell progression. This graph was then employed to order malignant cells along the pseudotime axis using the ordercells function within Monocle3. After ordering the cells, we plotted both the cells and the most differentially expressed stemness-related genes in pseudotime, adhering to the default parameters to visualize their expression patterns and dynamics across different stages of cell development. Furthermore, CytoTRACE (https://cytotrace.stanford.edu/) was utilized to assess the stemness and differentiation potential across various tumor cell subpopulations.

Bulk-seq data analysis

For the bulk-level analysis, we first filtered out samples that lacked survival information from the cohort. Subsequently, a survival analysis was conducted utilizing the Kaplan-Meier method. Univariate and multivariate Cox regression analyses were performed to identify independent risk factors that influence prognosis. These analyses were executed using the survival R package (https://github.com/therneau/survival).

Tumor potential immune therapy susceptibility analysis

TIDE is a computational tool[15, 16] designed to forecast the probability of cancer patients responding to immunotherapies (PD-1/CTLA4 checkpoint inhibitors) based on analysis of bulk sequencing data.

Cellchat analysis

Cell-cell signaling networks were systematically reconstructed using the CellChat algorithm[17], a computational framework designed for deconvolving microenvironmental crosstalk from single-cell transcriptomic data. The receptors related to SRGN pathway were manually obtained from previous studies[18-24] and added to the CellChat database.

Supplementary figures:

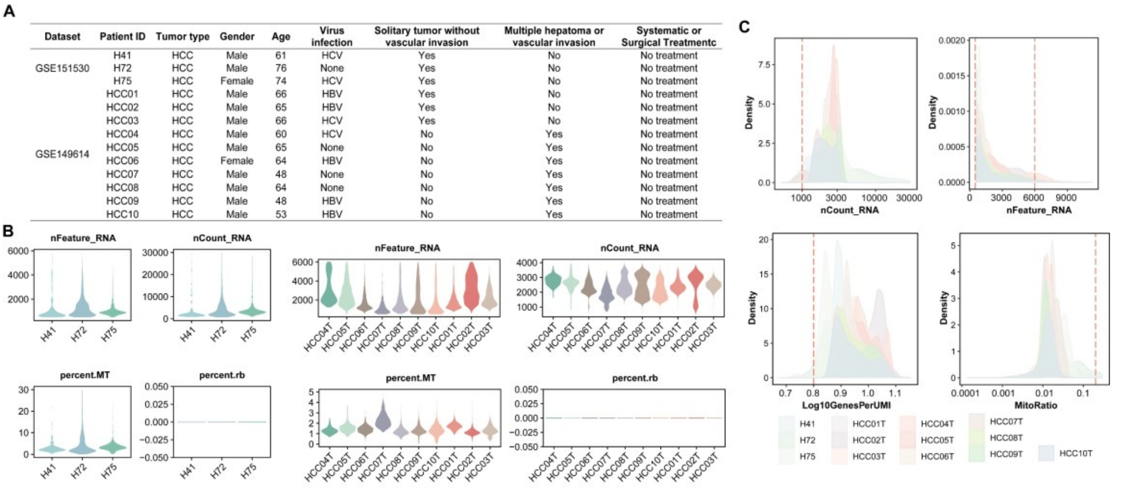

A

Dataset	Patient ID	Tumor type	Gender	Age	Virus infection	Solitary tumor without vascular invasion	Multiple hepatoma or vascular invasion	Systematic or Surgical Treatmentc
GSE151530	H41	HCC	Male	61	HCV	Yes	No	No treatment
	H72	HCC	Male	76	None	Yes	No	No treatment
	H75	HCC	Female	74	HCV	Yes	No	No treatment
GSE149614	HCC01	HCC	Male	66	HBV	Yes	No	No treatment
	HCC02	HCC	Male	65	HBV	Yes	No	No treatment
	HCC03	HCC	Male	66	HCV	Yes	No	No treatment
	HCC04	HCC	Male	60	HCV	No	Yes	No treatment
	HCC05	HCC	Male	65	None	No	Yes	No treatment
	HCC06	HCC	Female	64	HBV	No	Yes	No treatment
	HCC07	HCC	Male	48	None	No	Yes	No treatment
	HCC08	HCC	Male	64	None	No	Yes	No treatment
	HCC09	HCC	Male	48	HBV	No	Yes	No treatment
	HCC10	HCC	Male	53	HBV	No	Yes	No treatment

Supplementary Figure 1. Data quality control and filtering. (A) Clinical characteristics of the analyzed samples. (B) Violin plots showing the distribution of detected genes (nFeature RNA), total UMIs (nCount RNA), mitochondrial gene expression (percent MT), and ribosomal gene expression (percent rb) across samples. (C) Density plots illustrating the distribution of detected genes, UMIs, and MitoRatio per sample, with red lines indicating quality control thresholds.

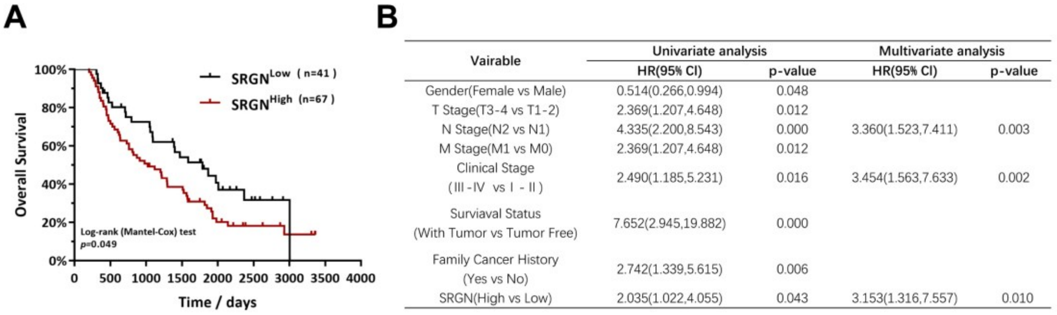

Vairable	Univariate analysis		Multivariate analysis	
	HR(95% CI)	p-value	HR(95% CI)	p-value
Gender(Female vs Male)	0.514(0.266,0.994)	0.048		
T Stage(T3-4 vs T1-2)	2.369(1.207,4.648)	0.012		
N Stage(N2 vs N1)	4.335(2.200,8.543)	0.000	3.360(1.523,7.411)	0.003
M Stage(M1 vs M0)	2.369(1.207,4.648)	0.012		
Clinical Stage (III-IV vs I-II)	2.490(1.185,5.231)	0.016	3.454(1.563,7.633)	0.002
Surviaval Status (With Tumor vs Tumor Free)	7.652(2.945,19.882)	0.000		
Family Cancer History (Yes vs No)	2.742(1.339,5.615)	0.006		
SRGN(High vs Low)	2.035(1.022,4.055)	0.043	3.153(1.316,7.557)	0.010

Supplementary Figure 2. Survival analysis between SRGN-high and SRGN-low patients. (A) SRGN level in the patients' serum was positively correlated overall survival. (B) SRGN expression is an independent survival outcome indicator for HCC patients.

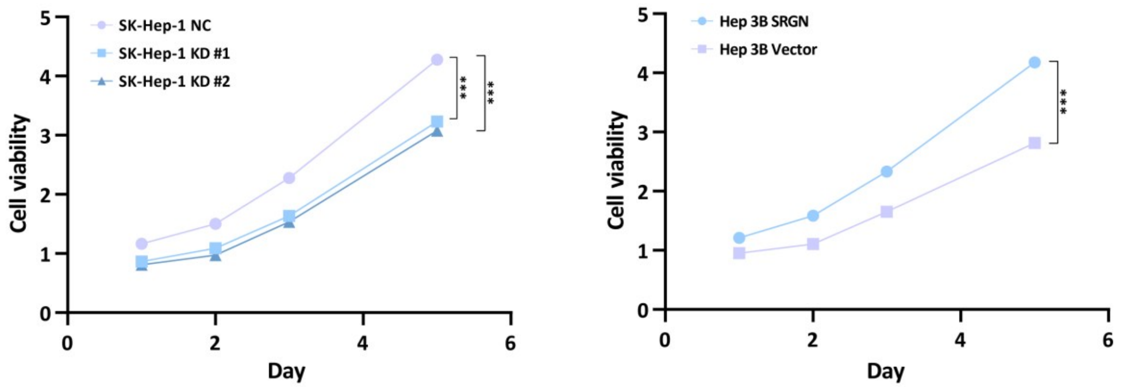

Supplementary Figure 3. MTS assays show that the cell viability of the SRGN-knockdown groups (SK-Hep-1 KD 1#, SK-Hep-1 KD 2#) is lower than that of the non-knockdown group (SK-Hep-1 NC), and the Hep 3B SRGN-overexpression group has higher cell viability than the Hep 3B vector group.

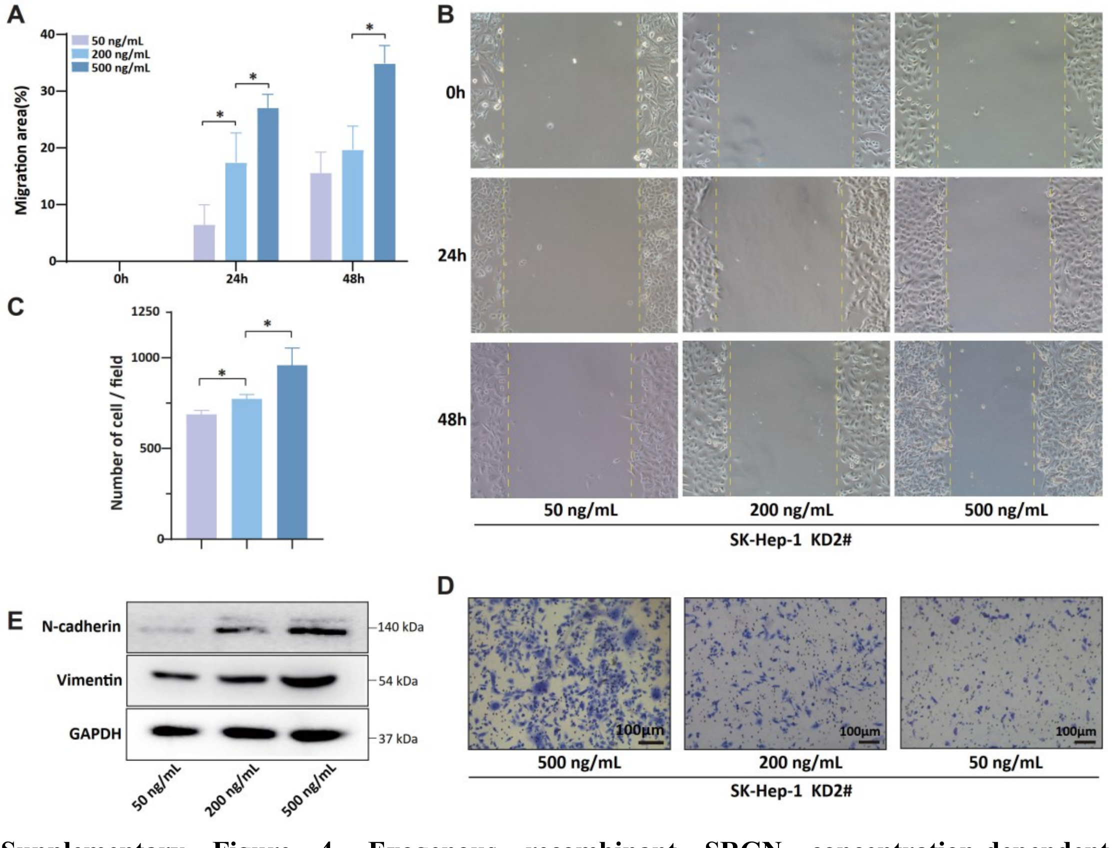

Supplementary Figure 4. Exogenous recombinant SRGN concentration-dependent enhancement of HCC Cell Migration and invasion via autocrine. (A) Quantifying wound closure area (%) in SK-Hep-1 KD2# cells treated with conditioned medium containing graded exogenous recombinant SRGN concentrations (50, 200, 500 ng/mL). (B) Phase-contrast images of wound healing assays at 0h, 24h, and 48h. (C) Quantifying invaded cells in Transwell chambers after 24 h exposure to SRGN-enriched conditioned medium. (D) Crystal violet-stained Transwell membranes demonstrating invasion capacity. (E) Immunoblot analysis of N-cadherin and vimentin level when HCC cells exposed to SRGN dose gradients. *p<0.05.

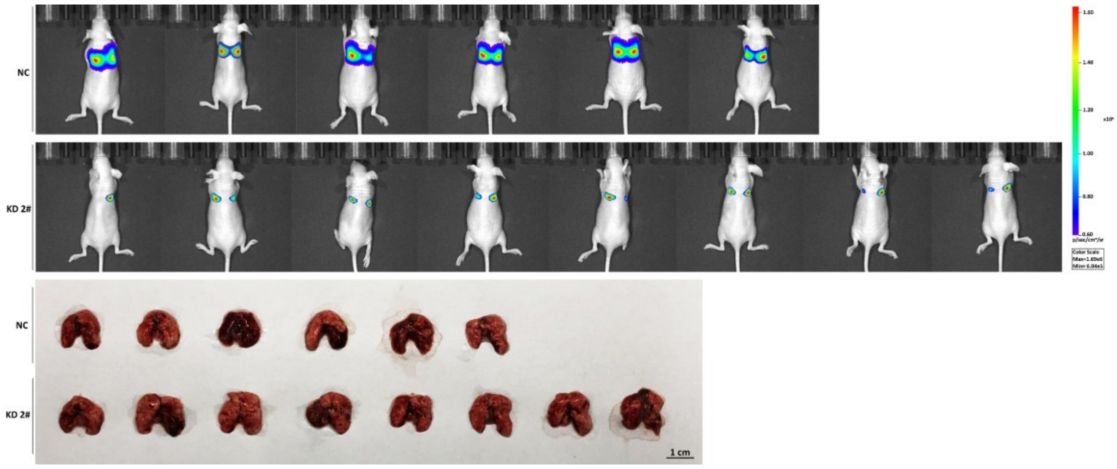

Supplementary Figure 5. In vivo, fluorescence imaging quantifies the burden and dissemination of HCC metastatic tumors in the lungs of the control group (NC) and the SRGN knockdown group (KD 2#) and anatomical sampling photos of lung tissue.

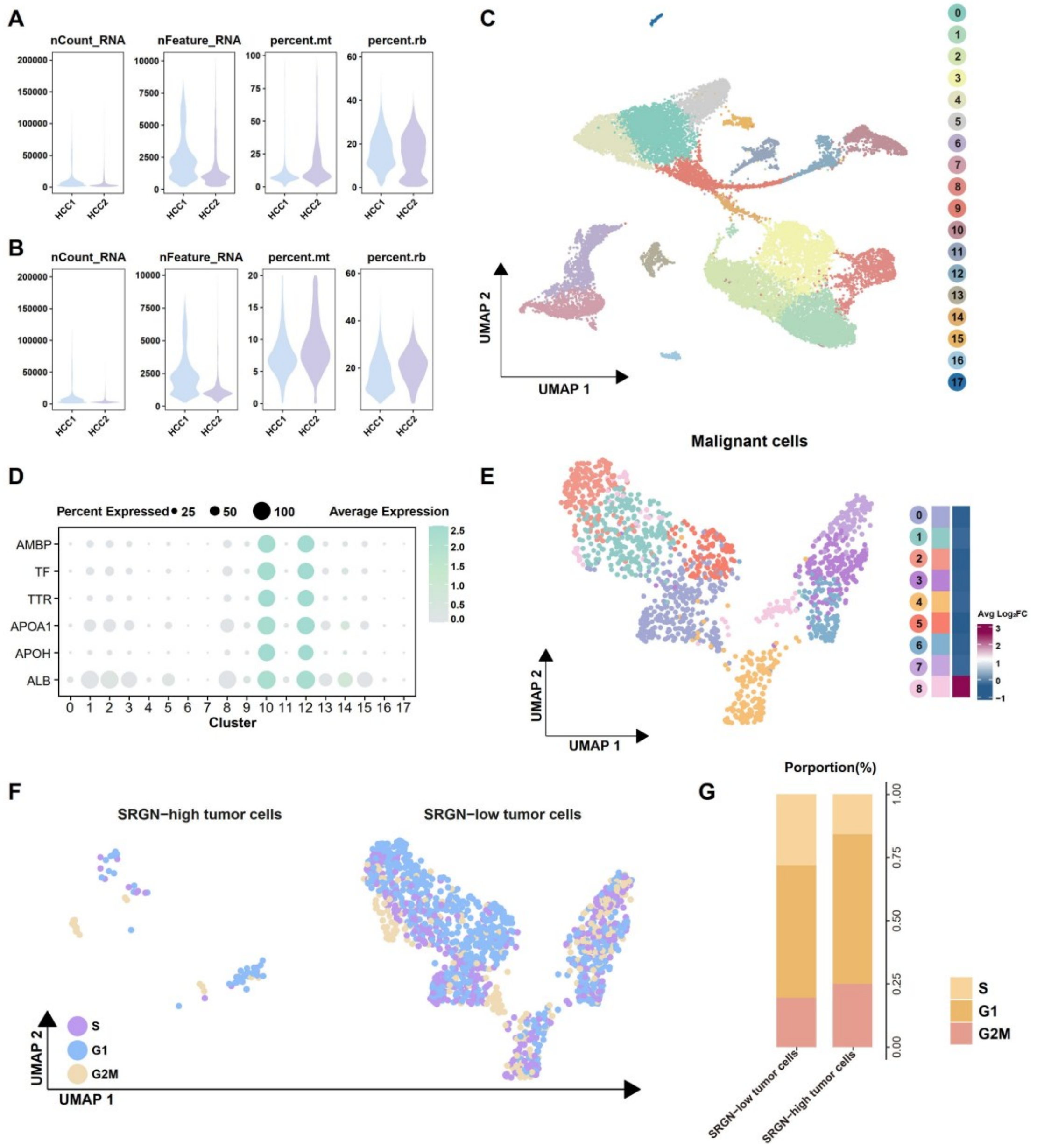

Supplementary Figure 6. Cell cycle phase distribution analysis between SRGN-high and SRGN-low tumor subclusters. (A) Pre-quality control of the GSE166635 cohort. (B) Post-quality control filtering (mitochondrial genes <5%). (C) UMAP dimensionality reduction with Leiden algorithm-derived clusters (resolution=0.5). (D) Dot plot of lineage-defining markers (AMBP, TF, TTR, APOA1, APOH, and ALB) for malignant subcluster identification. (E) Heatmap of SRGN expression across malignant subclusters UMAP, highlighting subcluster 8 as SRGN-high tumor cells. (F) Cell cycle phase projection (G1/S/G2M), stratified by SRGN expression. (G) A stacked bar plot comparing phase distribution showed more cells in G1 and G2M phases in SRGN-high tumor cells than in SRGN-low tumor cells.

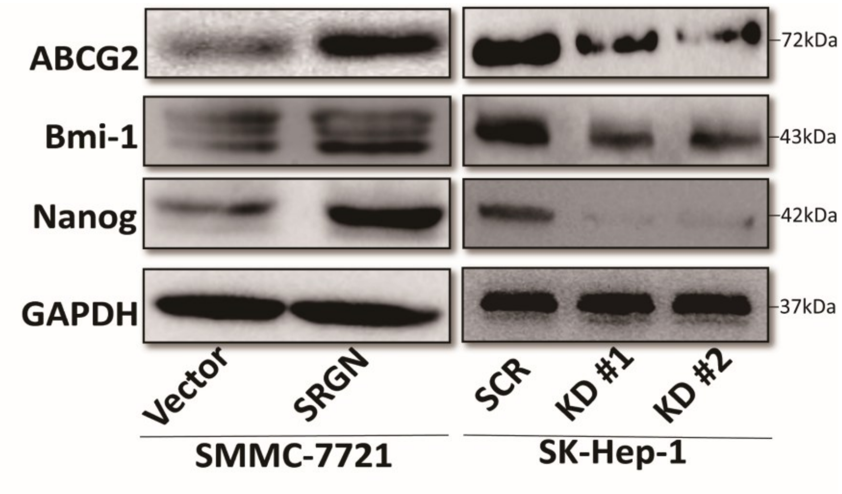

Supplementary Figure 7. Immunoblot analysis of cancer stem cell markers modulated by SRGN expression. SRGN overexpression in SMMC-7721 and SRGN knockdown (KD) SK-Hep-1 cells were performed, followed by immunoblotting for cancer stem cell-associated markers at 72 h post-transfection.

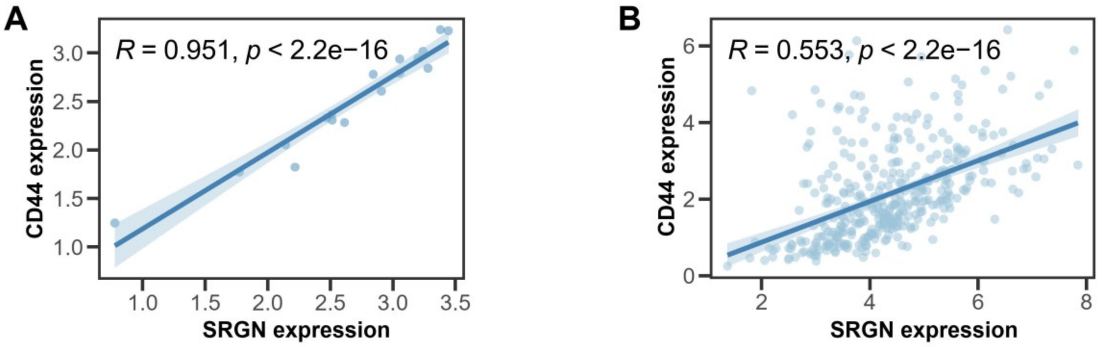

Supplementary Figure 8. Correlation analysis between SRGN and CD44 in HCC. (A)At single cell trancriptomic level, SRGN expression positively related with CD44 expression. (B) At bulk-seq level, SRGN expression positively related with CD44 expression in the LIHC corhort.

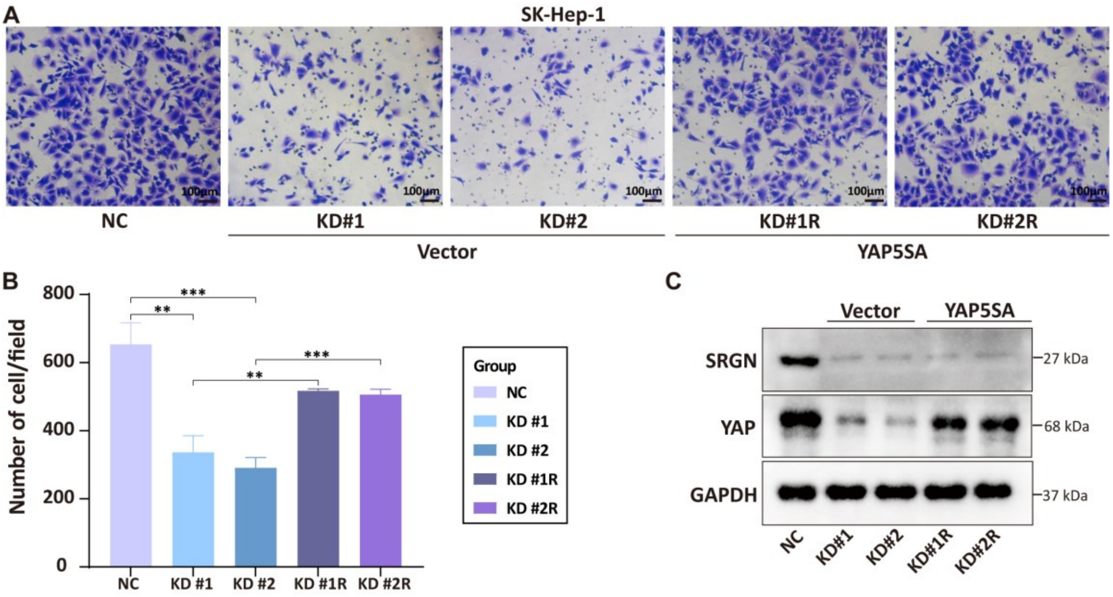

Supplementary Figure 9. SRGN regulates cell migration through YAP-dependent pathways. (A) Representative Transwell migration images. (B) Quantification of migrated cells. (C) Western blot analysis of YAP pathway activation. NC: SRGN non-knockdown control group. SRGN knockdown comparison group: KD1#, KD2#. YAP5SA rescue group: KD1#R, KD2#R. **: $p < 0.01$, ***: $p < 0.001$.

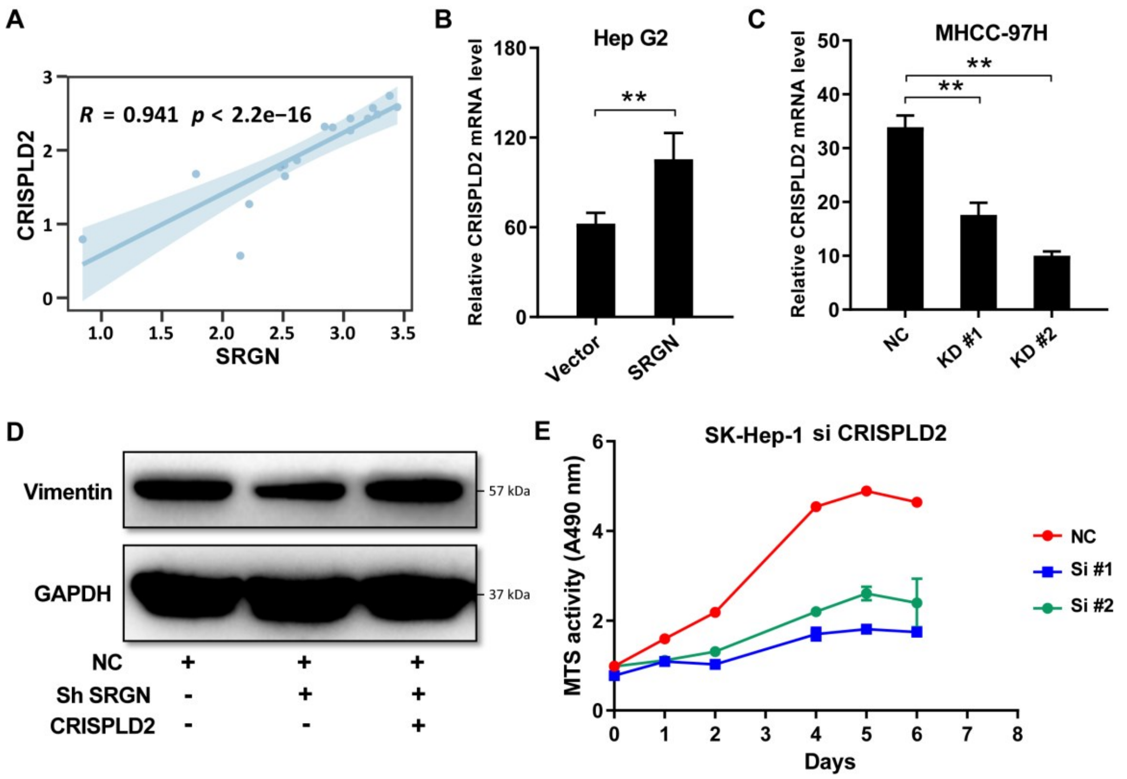

Supplementary Figure 10. Correlation analysis between SRGN and CRISPLD2 in HCC. (A) Positive correlation between SRGN and CRISPLD2 expression at the single-cell transcriptomic level. (B, C) Positive correlation between SRGN and CRISPLD2 expression in Hep-G2 and MHCC-97H cells. (D) Overexpression of CRISPLD2 increases vimentin levels in SRGN-knockdown SK-Hep-1 cells. (E) Suppression of CRISPLD2 significantly reduces proliferation in SK-Hep-1 cells.

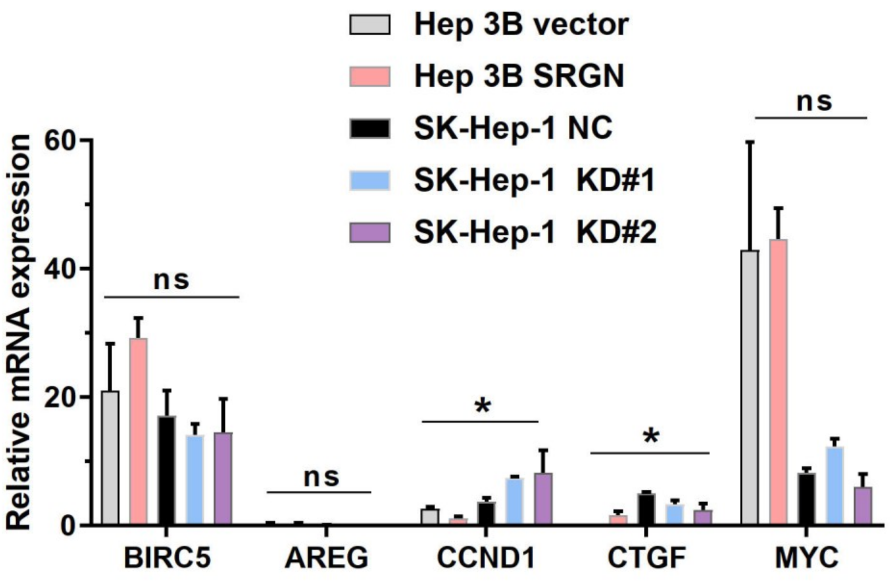

Supplementary Figure 11. qPCR for YAP/TEAD1 target genes in SRGN-overexpression and SRGN-knockdown HCC cells.

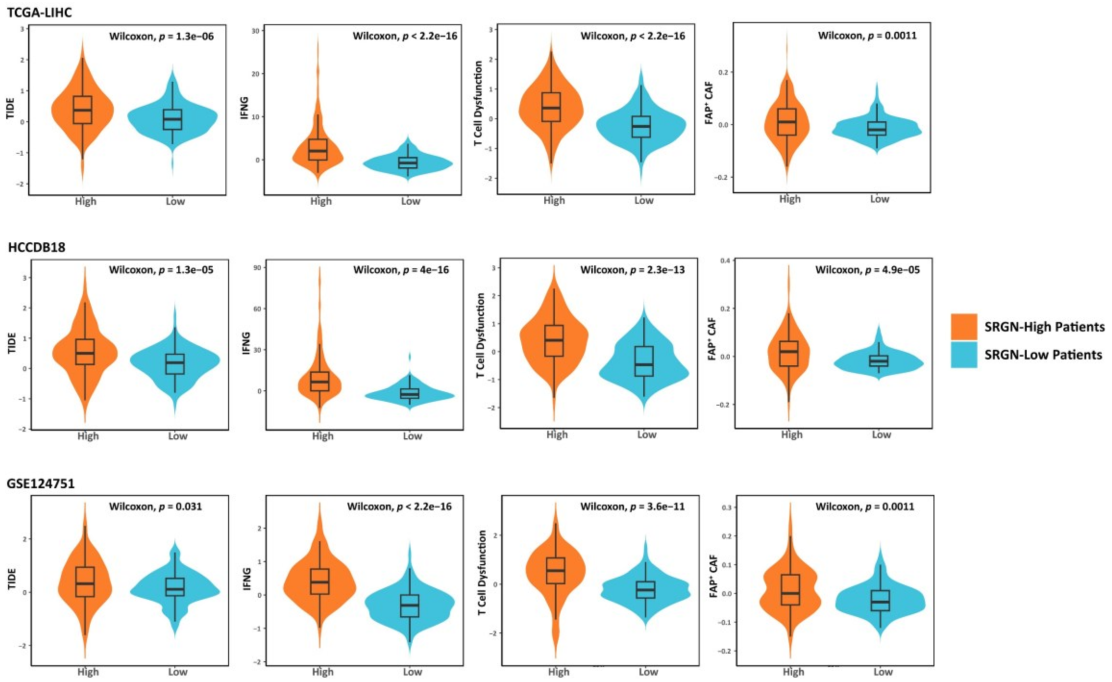

Supplementary Figure 12. Potential immune therapy susceptibility comparison between patients with high versus low SRGN expression HCC at the bulk-seq level of clinical cohorts. TIDE: High values indicate a higher potential of HCC immune evasion and less likely benefit from anti-PD1/CTLA1 therapy. IFGN: IFN-γ signature. T Cell Dysfunction: Scores show dysfunction of T cells in patients. FAP^{+}CAF: Correlation of patients and FAP^{+} CAF.

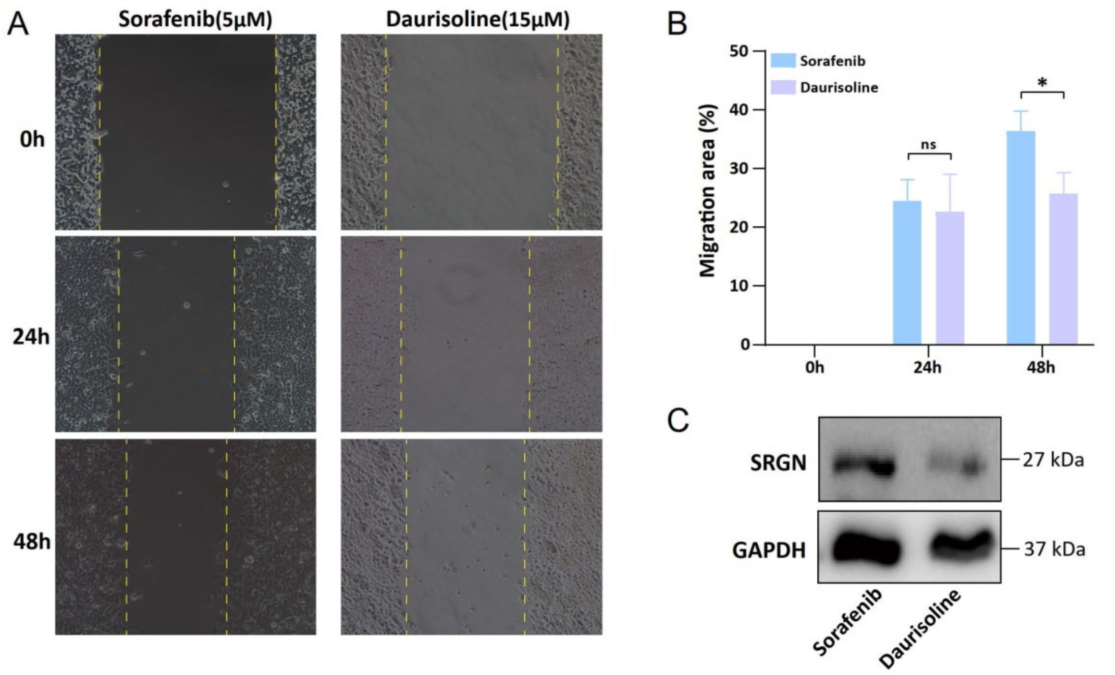

Supplementary Figure 13. Comparative analysis of sorafenib and daurisoline on migration inhibition and SRGN expression in MHCC-97H cells. (A) Wound healing assay images at 0h, 24h and 48h post-treatment. (B) Quantification of migration area. (C) Immunoblotting of SRGN protein levels after 48h drug exposure.

Supplementary tables:

Primer		Sequence
**SRGN**		
	Forward	5'-CGTCTGAGGACTGACCTTTTTCC-3'
	Reverse	5'-CGTTAGGAAGCCACTCCCAGAT-3'
**CRISPLD2**		
	Forward	5'-ACGGACGAGATGAATGAGGTGG-3'
	Reverse	5'-GGTGTCACATCTGACGACTTGG-3'

Supplementary Table 1. **Primes sequences used in this study.**

**Library 1: FDA Approved Drugs**			**Library 2: Traditional Chinese Medicine**		
Drug Bank Accession Number	Score	Generic Name	ID	Score	Generic Name
DB00762	-9.5	Irinotecan	T2912	-9.9	Ergosterol
DB13879	-9.3	Glecaprevir	T3054	-9.1	**Daurisoline**
DB00390	-9.3	Digoxin	T5S1028	-8.9	3,29-Dibenzoyl Rarounitriol
DB00941	-9	Hexafluronium	T4923	-8.8	7-Dehydrocholesterol
DB00696	-9	Ergotamine	T2775	-8.8	**Baicalin**
DB14703	-9	Dexamethasone metasulfobenzoate	TN1006	-8.6	Tirucallol
DB06210	-8.7	Eltrombopag	T2S0112	-8.6	Yibeinoside A
DB12371	-8.6	Siponimod	T7600	-8.5	Fucoxanthin
DB15328	-8.5	Ubrogepant	T0478	-8.5	Progesterone
DB01396	-8.4	Digitoxin	T2763	-8.4	Panaxadiol
DB00471	-8.4	Montelukast	T10993	-8.4	δ-Amyrenone
DB05812	-8.4	Abiraterone	T2972	-8.3	Rutaecarpine
DB00163	-8.4	Vitamin E	T3S1775	-8.3	Tectochrysin
DB00398	-8.4	**Sorafenib**	T3871	-8.2	Sitogluside
DB12887	-8.3	Tazemetostat	T6S1315	-8.2	Oroxylin A
DB08875	-8.3	Cabozantinib	T3S2027	-8.2	Rubusoside
DB00307	-8.2	Bexarotene	TN5031	-8.1	Sitostenone
DB00378	-8.2	Dydrogesterone	T6169	-8.1	Indirubin
DB11637	-8.2	Delamanid	T5S1103	-8.1	Isoliensinine
DB11363	-8.1	Alectinib			
DB01232	-8.1	Saquinavir			
DB08896	-8.1	**Regorafenib**			

**Library 3: Experimental Drugs**		
Drug Bank Accession Number	Score	Generic Name or ZINC
DB02729	-9.5	SD146
DB02702	-8.9	XV638
DB02354	-8.9	4-{[1-Methyl-5-(2-Methyl-Benzoimidazol-1-Ylmethyl)-1h-Benzoimidazol-2-Ylmethyl]-Amino}-Benzamidine
DB07181	-8.7	4'-[(1R)-1-amino-2-(2,5-difluorophenyl)ethyl]biphenyl-3-carboxamide
DB07728	-8.7	ZINC000016052794
DB07530	-8.7	ZINC000038911928
DB03159	-8.6	CRA_8696
DB06925	-8.6	3-(2-AMINOQUINAZOLIN-6-YL)-4-METHYL-N-[3-(TRIFLUOROMETHYL)PHENYL]BENZAMIDE
DB02915	-8.6	4-(2,4-Dimethyl-1,3-thiazol-5-yl)-N-[4-(trifluoromethyl)phenyl]-2-pyrimidinamine
DB03373	-8.6	ZK-806711
DB08191	-8.5	4-(5-phenyl-1H-pyrrolo[2,3-b]pyridin-3-yl)benzoic acid

DB08121	-8.5	(2S)-2-(biphenyl-4-yloxy)-3-phenylpropanoic acid
DB01725	-8.5	2-{2-hydroxy-[1,1'-biphenyl]-3-yl}-1H-1,3-benzodiazole-5-carboximidamide
DB13484	-8.5	Quinbolone
DB07837	-8.5	[4-(5-naphthalen-2-yl-1H-pyrrolo[2,3-b]pyridin-3-yl)phenyl]acetic acid
DB14070	-8.5	HM-30181
DB07778	-8.4	(S)-famoxadone
DB04578	-8.3	ZINC000012504428
DB08591	-8.3	5-(4-METHOXYBIPHENYL-3-YL)-1,2,5-THIADIAZOLIDIN-3-ONE 1,1-DIOXIDE
DB04204	-8.3	[(4-{4-[4-(Difluoro-Phosphono-Methyl)-Phenyl]-Butyl}-Phenyl)-Difluoro-Methyl]-Phosphonic Acid
DB14035	-8.2	Englitazone
DB01443	-8.2	19-Nor-5-androstenedione
DB07151	-8.2	4-(4-hydroxy-3-methylphenyl)-6-phenylpyrimidin-2(5H)-one
DB07080	-8.2	TO-901317
DB08025	-8.2	N-{2'-[(4-FLUOROPHENYL)AMINO]-4,4'-BIPYRIDIN-2-YL}-4-METHOXYCYCLOHEXANECARBOXAMIDE
DB06944	-8.2	N-(3-cyclopropyl-1H-pyrazol-5-yl)-2-(2-naphthyl)acetamide
DB07827	-8.1	ZINC000003815953
DB03571	-8.1	3-(5-amino-7-hydroxy-[1,2,3]triazolo[4,5-d]pyrimidin-2-yl)-N-(3,5-dichlorobenzyl)-benzamide
DB03865	-8.1	6-Chloro-2-(2-Hydroxy-Biphenyl-3-Yl)-1h-Indole-5-Carboxamidine
DB06997	-8.1	2-(4-fluorophenyl)-N-{[3-fluoro-4-(1H-pyrrolo[2,3-b]pyridin-4-yloxy)phenyl]carbamoyl}acetamide
DB08036	-8.1	6,7,12,13-tetrahydro-5H-indolo[2,3-a]pyrrolo[3,4-c]carbazol-5-one
DB07642	-8.1	5-{[1-(2-fluorobenzyl)piperidin-4-yl]methoxy}quinazoline-2,4-diamine
DB13857	-8.1	Demegestone

Supplementary Table 2. Drug screening results targeting SRGN protein of HCC.

Supplementary references:

1. Chiang DY, Villanueva A, Hoshida Y, Peix J, Newell P, Minguez B, et al. Focal gains of VEGFA and molecular classification of hepatocellular carcinoma. Cancer Res. 2008; 68: 6779-88.doi: 10.1158/0008-5472.CAN-08-0742.
2. Anastassiou D, Rumjantseva V, Cheng W, Huang J, Canoll PD, Yamashiro DJ, et al. Human cancer cells express Slug-based epithelial-mesenchymal transition gene expression signature obtained in vivo. BMC Cancer. 2011; 11: 529.doi: 10.1186/1471-2407-11-529.
3. Kim SH, Turnbull J, Guimond S. Extracellular matrix and cell signalling: the dynamic cooperation of integrin, proteoglycan and growth factor receptor. J Endocrinol. 2011; 209: 139-51.doi: 10.1530/JOE-10-0377.
4. Gordon MK, Hahn RA. Collagens. Cell Tissue Res. 2010; 339: 247-57.doi: 10.1007/s00441-009-0844-4.
5. Alghamdi N, Chang W, Dang P, Lu X, Wan C, Gampala S, et al. A graph neural network model to estimate cell-wise metabolic flux using single-cell RNA-seq data. Genome Res. 2021; 31: 1867-84.doi: 10.1101/gr.271205.120.
6. Kanehisa M, Goto S. KEGG: kyoto encyclopedia of genes and genomes. Nucleic Acids Res. 2000; 28: 27-30.doi: 10.1093/nar/28.1.27.
7. Fan C, Chen F, Chen Y, Huang L, Wang M, Liu Y, et al. irGSEA: the integration of single-cell rank-based gene set enrichment analysis. Brief Bioinform. 2024; 25.doi: 10.1093/bib/bbae243.
8. Aibar S, Gonzalez-Blas CB, Moerman T, Huynh-Thu VA, Imrichova H, Hulselmans G, et al. SCENIC: single-cell regulatory network inference and clustering. Nat Methods. 2017; 14: 1083-6.doi: 10.1038/nmeth.4463.
9. DeTomaso D, Jones MG, Subramaniam M, Ashuach T, Ye CJ, Yosef N. Functional interpretation of single cell similarity maps. Nat Commun. 2019; 10: 4376.doi: 10.1038/s41467-019-12235-0.
10. Hanzelmann S, Castelo R, Guinney J. GSVA: gene set variation analysis for microarray and RNA-seq data. BMC Bioinformatics. 2013; 14: 7.doi: 10.1186/1471-2105-14-7.
11. Foroutan M, Bhuva DD, Lyu R, Horan K, Cursons J, Davis MJ. Single sample scoring of molecular phenotypes. BMC Bioinformatics. 2018; 19: 404.doi: 10.1186/s12859-018-2435-4.
12. Barbie DA, Tamayo P, Boehm JS, Kim SY, Moody SE, Dunn IF, et al. Systematic RNA interference reveals that oncogenic KRAS-driven cancers require TBK1. Nature. 2009; 462: 108-12.doi: 10.1038/nature08460.
13. Noureen N, Ye Z, Chen Y, Wang X, Zheng S. Signature-scoring methods developed for bulk samples are not adequate for cancer single-cell RNA sequencing data. eLife. 2022; 11.doi: 10.7554/eLife.71994.
14. Kolde R, Laur S, Adler P, Vilo J. Robust rank aggregation for gene list integration and meta-analysis. Bioinformatics. 2012; 28: 573-80.doi: 10.1093/bioinformatics/btr709.
15. Jiang P, Gu S, Pan D, Fu J, Sahu A, Hu X, et al. Signatures of T cell dysfunction and exclusion predict cancer immunotherapy response. Nat Med. 2018; 24: 1550-8.doi: 10.1038/s41591-018-0136-1.
16. Fu J, Li K, Zhang W, Wan C, Zhang J, Jiang P, et al. Large-scale public data reuse to model immunotherapy response and resistance. Genome Med. 2020; 12: 21.doi: 10.1186/s13073-020-0721-z.
17. Jin S, Guerrero-Juarez CF, Zhang L, Chang I, Ramos R, Kuan CH, et al. Inference and analysis

of cell-cell communication using CellChat. Nat Commun. 2021; 12: 1088.doi: 10.1038/s41467-021-21246-9.
18. Zhang Z, Qiu N, Yin J, Zhang J, Liu H, Guo W, et al. SRGN crosstalks with YAP to maintain chemoresistance and stemness in breast cancer cells by modulating HDAC2 expression. Theranostics. 2020; 10: 4290-307.doi: 10.7150/thno.41008.
19. Lim J, Hao T, Shaw C, Patel AJ, Szabo G, Rual JF, et al. A protein-protein interaction network for human inherited ataxias and disorders of Purkinje cell degeneration. Cell. 2006; 125: 801-14.doi: 10.1016/j.cell.2006.03.032.
20. Johnson JM, Castle J, Garrett-Engele P, Kan Z, Loerch PM, Armour CD, et al. Genome-wide survey of human alternative pre-mRNA splicing with exon junction microarrays. Science. 2003; 302: 2141-4.doi: 10.1126/science.1090100.
21. Bild AH, Yao G, Chang JT, Wang Q, Potti A, Chasse D, et al. Oncogenic pathway signatures in human cancers as a guide to targeted therapies. Nature. 2006; 439: 353-7.doi: 10.1038/nature04296.
22. Chen X, Cheung ST, So S, Fan ST, Barry C, Higgins J, et al. Gene expression patterns in human liver cancers. Mol Biol Cell. 2002; 13: 1929-39.doi: 10.1091/mbc.02-02-0023.
23. Wu G, Feng X, Stein L. A human functional protein interaction network and its application to cancer data analysis. Genome Biol. 2010; 11: R53.doi: 10.1186/gb-2010-11-5-r53.
24. Innocenti F, Cooper GM, Stanaway IB, Gamazon ER, Smith JD, Mirkov S, et al. Identification, replication, and functional fine-mapping of expression quantitative trait loci in primary human liver tissue. PLoS Genet. 2011; 7: e1002078.doi: 10.1371/journal.pgen.1002078.
